# Functional-proteomics-based investigation of the cellular response to farnesyltransferase inhibition in lung cancer

**DOI:** 10.1016/j.isci.2025.111864

**Published:** 2025-01-21

**Authors:** Yanbo Pan, Olena Berkovska, Soumitra Marathe, Georgios Mermelekas, Greta Gudoityte, Amare D. Wolide, Taner Arslan, Brinton Seashore-Ludlow, Janne Lehtiö, Lukas M. Orre

**Affiliations:** 1 SciLifeLab, Department of Oncology and Pathology, Karolinska Institutet, 17165 Solna, Sweden

**Keywords:** Cell biology, Cancer, Proteomics

## Abstract

Farnesylation is a lipid post-translational modification of proteins crucial for protein membrane anchoring and cellular signaling. Farnesyltransferase inhibitors (FTIs), such as tipifarnib, are being tested in cancer therapy. However, the full impact of FTIs on farnesylation substrates remains poorly understood, thus limiting their use in precision medicine. In this study, we performed a global proteomics analysis to investigate farnesylation and the effects of tipifarnib in lung cancer cell lines. Using metabolic labeling and mass spectrometry, we identified farnesylated proteins and mapped their subcellular localization. We also analyzed tipifarnib-dependent protein relocalization and proteome-wide changes. Key findings include the potential therapeutic value of FTIs for NRAS-mutated melanoma and GNAQ/GNA11-mutated uveal melanoma by inhibiting INPP5A farnesylation. Additionally, we identified a synergistic drug combination involving tipifarnib and a ferroptosis inducer and discovered PTP4A1 as a regulator of interferon signaling. Our data, covering 15,080 proteins, offer valuable insights for future studies of farnesylation and FTIs.

## Highlights

• Proteome-wide identification of prenylated/farnesylated proteins and their localization
• Protein relocalization analysis after tipifarnib treatment covering > 12,000 proteins
• In-depth profiling of tipifarnib effects on the proteome of five lung cancer cell lines
• Accessible resource combining all generated data

## Graphical abstract

**Figure:**
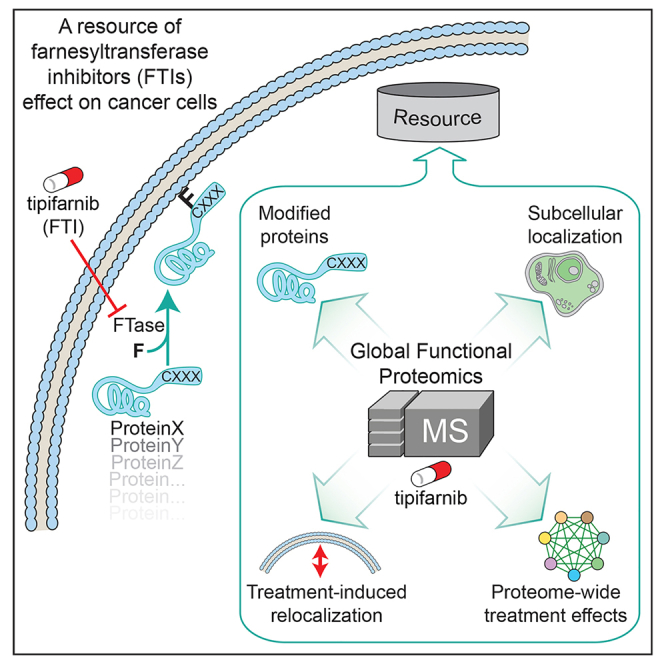

## Introduction

Farnesylation is a type of protein post-translational modification (PTM) in which farnesyltransferase (FTase) attaches a farnesyl group, a 15-carbon isoprenoid lipid, to a cysteine near the C-terminus of a protein. A similar PTM involving a 20-carbon lipid is called geranylgeranylation, and together with farnesylation, they are known as prenylation. These modifications facilitate protein localization by intracellular anchoring of the protein to membranes and may impact specific protein-protein interactions and/or protein stability.^1^ An extensively studied example of proteins with prenylation-dependent function are the oncogenic Ras family proteins.^2^ The three Ras genes (KRAS, NRAS, and HRAS) are frequently mutated in cancer (11%, 3%, and 1% of cancers in the US, respectively),^3^ and the corresponding proteins are active when localized to the membrane.^4^ Consequently, pre-clinical and clinical development of FTase inhibitors (FTIs) was initiated in 1990s to inhibit the farnesyl-dependent membrane anchoring and prevent the proteins’ activity.^2^ Two inhibitors (lonafarnib and tipifarnib) advanced to phase III clinical trials in several solid tumor types.^5^^,^^6^^,^^7^ Both drugs have acceptable toxicity profiles, but disappointingly, neither of the drugs showed overall survival benefit in the investigated cancers.^5^^,^^6^^,^^7^^,^^8^ It was then shown that KRAS and NRAS can alternatively be geranylgeranylated, whereas HRAS is specifically dependent on farnesylation.^9^ Therefore, clinical development of tipifarnib continued with a focus on HRAS-mutant head and neck squamous cell carcinoma (HNSCC).^10^ Furthermore, preclinical studies have provided rationale for drug combinations using FTIs with other targeted therapies. For example, tipifarnib was shown to be effective in PIK3CA/HRAS-dysregulated HNSCC when combined with a PI3Kα inhibitor, alpelisib.^11^ The synergy was explained by a sustained mammalian target of rapamycin (mTOR) inhibition via inhibition of farnesylation of both HRAS and RHEB, another FTase substrate. This finding highlights the importance of FTase substrates beyond Ras family proteins. Moreover, a next-generation FTI, KO-2806, has re-opened a possible indication in KRAS-mutant cancers. KO-2806 has been shown to improve efficacy of KRAS^G12C^ inhibitor adagrasib by blocking compensatory bypass mechanisms of KRAS inhibition in KRAS-mutant non-small cell lung cancer (NSCLC).^12^ As of 2024, the FTI-based drug combinations are in active phase 1/2 clinical trials in advanced solid tumors. Furthermore, Food and Drug Administration (FDA) has granted tipifarnib a breakthrough therapy designation,^13^ highlighting a clear potential for FTIs in the treatment of cancer. In addition, extending to non-oncological indications, lonafarnib was recently approved for the use in the rare pediatric premature aging disorder Hutchinson-Gilford progeria syndrome (HGPS) to prevent farnesylation of mutant lamin A/C gene (LMNA).^14^ However, identifying relevant patient groups and effective drug combinations remains a complex task due to the lack of knowledge on proteome-wide farnesylation targets, their farnesylation-dependent (re)localization, and how they intertwine with other cellular signaling.

Previously, it was believed that FTase requires a C-terminal CaaX motif where C is a cysteine, aa, two aliphatic amino acids, and X, any amino acid. However, more recent studies have shown that FTase has broader reactivity across a majority of CXXX sequences.^15^ Based on this definition of a farnesylation motif, there may be more than 2,000 farnesylation substrates, 92 of which have been experimentally validated in cell lines (CLs)^16^^,^^17^^,^^18^^,^^19^^,^^20^ and 388 more in peptide-based assays.^21^^,^^22^^,^^23^ Furthermore, at least seven proteins without a CXXX motif were found to be farnesylated in human CLs.^18^ Previous research aiming to identify farnesylated proteins has generated tools for enrichment of these proteins, but so far, these methods have not been coupled with farnesylation-dependent localization analyses or in-depth evaluation of the downstream phenotypic effects.

**Figure 1:**
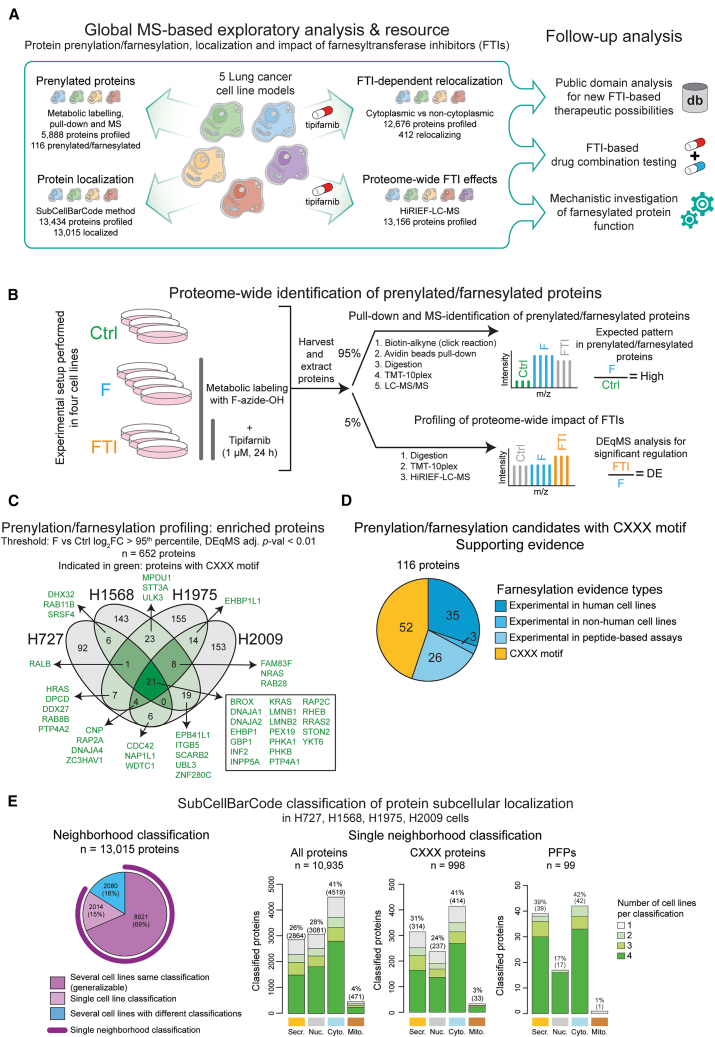
Identification of prenylated/farnesylated proteins and protein localization profiling in lung cancer cell lines (A) Schematic study overview. (B) Experimental design for identification of prenylated/farnesylated proteins (PFPs). F-azide-OH, farnesyl alcohol azide; FTI, farnesyltransferase inhibitor; TMT, tandem mass tag; HiRIEF-LC-MS, high-resolution isoelectric focusing-liquid chromatography-mass spectrometry. (C) Enriched proteins in the F-azide-tagged pull-down. (D) Farnesylation supporting evidence for enriched proteins that have a CXXX motif. (E) Subcellular localization classification using the SubCellBarCode method. Number of classified proteins across the cell lines (left); neighborhood classification (Secr., secretory; Nuc., nucleus; Cyto., cytosol; Mito., mitochondria) for proteins with a single neighborhood classification, i.e., no conflicting classification in different cell lines (right). In panels (C) and (E), the NCI- prefix has been omitted in the cell line names.

Here, we performed proteome-wide identification of prenylated/farnesylated proteins in multiple CL models by metabolic farnesyl labeling followed by click-chemistry-facilitated affinity purification with in-depth mass spectrometry (MS)-based proteomics. We complemented the findings with analyses based on our previously developed SubCellBarCode method to evaluate the impact of prenylation on protein subcellular localization. Further, we investigated the impact of farnesyltransferase inhibition on protein localization in lung cancer cells using a here-developed simplified method for global protein relocalization analysis. Finally, we performed in-depth proteomics profiling to investigate the global effects of farnesyltransferase inhibition on the cellular phenotype and signaling. Combined, this data provide a comprehensive resource for future studies of farnesylation and the impact of farnesyl transferase inhibitors in cancer cells (Figure 1A).

Based on the generated data, we performed follow-up investigations including *in vitro* experiments and public domain data analysis. Therewith, we reveal the functional role of a farnesylation substrate PTP4A1, a new synergistic drug combination of an FTI and a ferroptosis-inducing drug, and new cancer subgroups that may benefit from FTI treatment.

## Results

### Proteome-wide identification of protein prenylation in lung cancer CLs

To expand the knowledge of protein prenylation/farnesylation in cancer, and lung cancer in particular, we performed MS-based proteomic analysis of prenylated/farnesylated proteins (PFPs) and their localization in cancer CLs. For this purpose, we selected four lung cancer CLs that, according to the Genomics of Drug Sensitivity in Cancer (GDSC) drug screening dataset,^24^ respond to an FTI, tipifarnib (NCI-H1568, NCI-H1975, NCI-H727, and NCI-H2009; Figure S1A).

For the identification of PFPs, we used metabolic labeling for PFP pull-down coupled with our in-depth proteomics method, HiRIEF LC-MS^25^ (Figure 1B). A metabolic label, azido farnesyl alcohol (F-azide-OH), is efficiently used in endogenous FTase reactions, resulting in tagging of FTase substrate proteins.^20^ After extraction of total protein lysates, the tag was used for click-chemistry-based enrichment of modified proteins. Namely, a reaction between biotin alkyne and F-azide-tagged proteins enabled their pull-down and identification using biotin-avidin affinity chromatography followed by MS. It should be noted that even though F-azide-OH is an analogue of the endogenous substrate of FTase FPP, it has been shown that this probe can be used by different prenylation enzymes.^26^ For that reason, enriched proteins are treated here as PFP candidates. In parallel, total protein lysates were also analyzed to profile the global impact of tipifarnib on the proteome 24 h after treatment, which will be discussed in a following section.

For stringent analysis of the generated data, the proteins identified in the pull-down experiment were filtered based on the ratio between F-azide-treated and control samples, where only the top 5% were kept. This cutoff was based on the evaluation of density distributions for all proteins and for proteins with a predicted farnesylation motif, CXXX (Figure S1C). In addition, we applied our previously developed statistical method for improved accuracy in MS data analysis, DEqMS^27^ (adjusted [adj.] *p* value <0.01), resulting in the identification of 652 enriched proteins (Figures 1C and S1C; Tables S2 and S3). Out of these enriched proteins, 116 have a CXXX motif and will be referred to as PFPs. Out of these, 64 have previous supporting evidence based on farnesylation experimental data,^16^^,^^17^^,^^18^^,^^19^^,^^20^^,^^21^^,^^22^^,^^23^ whereas 81 proteins have not been previously verified as farnesylation targets in human-cell-line-based experiments (Figure 1D; Table S3). A substantial number of proteins without the CXXX motif was also pulled down in the experiment, indicating that these proteins could either be interaction partners of PFPs, could have an alternative motif for FTase, or showed non-specific binding in the pull-down experiments (Figures S1D and S1E). It should be noted, the proportion of each FTase substrate that is farnesylated may depend on the cell type and cell state.^26^ Such variability combined with limits in analytical sensitivity can explain why we do not identify all PFPs in all CLs. For example, the known farnesylation substrates and kinetochore proteins CENPE and CENPF met our stringent cutoff for PFPs but were only identified in one CL each. On the other hand, HRAS and RHEB were identified in all four CLs, but HRAS only exceeded the stringent threshold in two of the CLs (Figure S1F).

Considering the role prenylation plays in protein subcellular localization, we also performed a proteome-wide protein localization analysis using our recently developed SubCellBarCode method (SCBC).^28^^,^^29^ This analysis resulted in localization information for 9,629–10,551 proteins per CL (Figures 1E and S1G–S1J; Tables S1 and S4). Importantly, this analysis indicated that the PFPs were, as expected, more frequently localized to membrane compartments (secretory neighborhood) compared to CXXX-motif proteins in general (Figure 1E). Interestingly, a substantial number of PFPs showed an overall cytosolic localization. This finding indicates that only a minor fraction of these proteins is prenylated in the cell, potentially due to regulated prenylation or expression of variants without the C-terminal prenylation motif.

To further investigate the differential localization among CXXX-motif proteins and PFPs, we performed a motif sequence analysis of the last four amino acids at the C-terminal (X_0_X_1_X_2_X_3_) (Figure S1B). Enrichment analysis comparing the amino acids in positions 1–3 in PFPs vs. all the proteins in the neighborhood-localized dataset (*n* = 13,015) revealed that aliphatic amino acids isoleucine (I) and leucine (L) were significantly enriched in proteins localized in the secretory neighborhood (log_2_ fold enrichments of 2.5 and 2.0 at position X_2_ and X_3_, respectively, with *q* values of 8.7 × 10^−4^ and 1.1 × 10^−5^, respectively). On the other hand, only isoleucine was also enriched at position X_2_ in the cytosolic neighborhood proteins. Instead, glutamine (Q) with an uncharged polar side chain was enriched at position X_3_ (log_2_ fold enrichment 3.1, q value 2.3 × 10^−10^). These differences between C-terminal motifs in the secretory and cytosolic neighborhoods were observed in PFPs identified in this study but not in the broader subset of proteins defined by the CXXX motif. Therefore, our analysis indicates that subcellular localization of prenylation substrates is determined not only by the ability to be prenylated but also by the C-terminal motif itself.

In summary, our analysis identified a large number of PFPs in lung cancer cells. These results support previous studies that show widespread use of prenylation in the proteome and indicate that pharmacological targeting of these modifications could have broad impact on the proteome. The activity of clinically promising farnesyl transferase inhibitors (FTI) is considered to rely on altering the localization of farnesylated proteins; therefore, we continued by investigating treatment-induced relocalizations.

### Proteome-wide analysis of farnesylation-dependent relocalization in lung cancer cells

**Figure 2:**
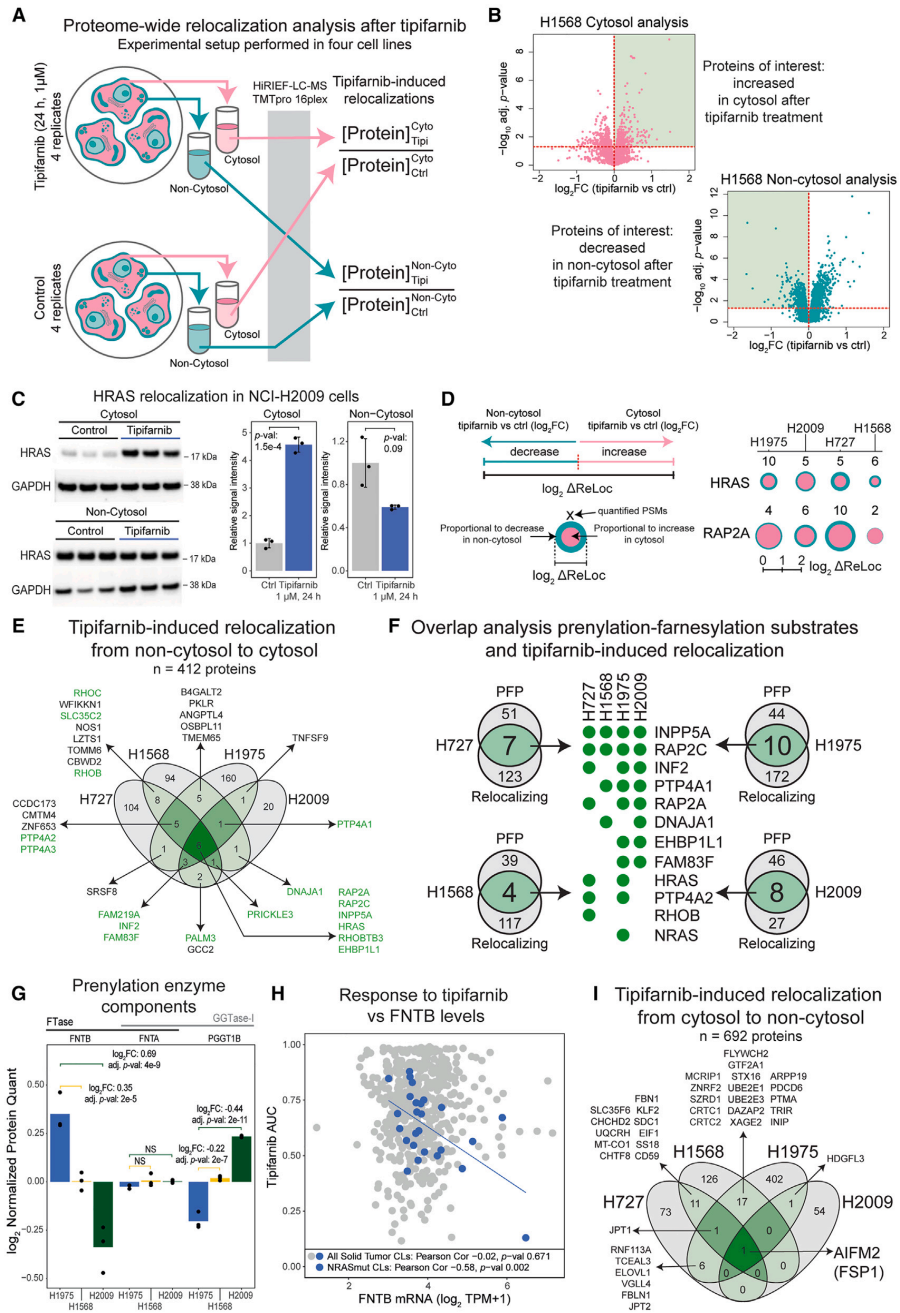
Tipifarnib induces widespread protein relocalization in lung cancer cell lines (A) Experimental design. HiRIEF-LC-MS, high-resolution isoelectric focusing-liquid chromatography-mass spectrometry; TMT, tandem mass tag; Tipi, tipifarnib; Cyto, cytosol; Non-Cyto, non-cytosol. (B) Approaches to identifying relocalizing proteins exemplified by data from NCI-H1568 cells. Fold change (FC) and adjusted *p* values (adj. *p*-val) were calculated using the DEqMS method. (C) Western blot and densitometry analysis of HRAS in cytosolic and non-cytosolic fractions with and without tipifarnib treatment (1 μM, 24 h) in NCI-H2009 cells. Densitometric values were normalized to GAPDH and then the control mean and are represented as mean ± SD (*n* = 3 independent cell cultures). *p* values (*p*-val) were calculated using Welch’s two-sided t test. (D) Quantification of protein relocalization using a delta ReLoc score (left) exemplified for HRAS and RAP2A (right). (E) Proteins relocalizing from the non-cytosol to the cytosol upon tipifarnib treatment (1 μM, 24 h). Criteria: log_2_ ΔReLoc >0.4, minimum DEqMS adj. *p*-val <0.05, opposite direction in cytosolic and non-cytosolic fractions. Proteins indicated in green have a CXXX motif. (F) Overlapping candidate farnesylation substrates (see Figure 1C) and proteins relocalizing upon tipifarnib treatment (see Figure 2E) for each cell line. (G) Protein levels of farnesyltransferase (FTase) and geranylgeranyltransferase type I (GGTase-I) components FNTB, FNTA, and PGGT1B as quantified by MS proteomics in the control samples from the 48-h tipifarnib treatment experiment (see below), represented as log_2_ geometric mean (*n* = 3 independent cell cultures). Fold change (FC) and adjusted *p* values (adj. *p*-val) were calculated using the DEqMS method. (H) FNTB mRNA levels (CCLE 21Q4 release) versus tipifarnib AUC (GDSC1 dataset) in all solid tumor cell lines (*n* = 531) and NRAS-mutant (mut) cell lines indicated in blue (*n* = 26, per Sanger Institute’s Cell Model Passport annotation). The associated Pearson’s correlation coefficient (Pearson Cor.) and two-sided *p* value (*p*-val) from t-distribution with *n −* 2 d.f. are provided. The regression line in blue corresponds to the NRASmut cell lines. (I) Proteins relocalizing from the cytosol to the non-cytosol upon tipifarnib treatment (1 μM, 24 h). Criteria: like for panel (E), but log_2_ ΔReLoc < −0.4. In panels, (B), (D–G), and (I), the NCI- prefix has been omitted in the cell line names.

Due to the impact of farnesylation on protein localization, we next investigated farnesylation-dependent protein relocalization in the same four lung cancer CLs in which we performed the prenylation/farnesylation profiling described earlier. To perturb protein farnesylation, we used tipifarnib, an FTI currently being tested in clinical trials for multiple cancers. We previously showed how the SCBC method can be applied for global analysis of protein relocalization by investigating the effects of an EGFR inhibitor on the spatial organization of the cellular proteome.^28^ Our studies indicated that protein relocalizations in the cell commonly take place to or from the cytosol. Our interpretation is that the cytosol, in such cases, is used as a reservoir, and when needed, proteins can be recruited to specific subcellular locations from this reservoir. Building further on these methods, we have here developed a simplified approach for proteome-wide analysis of relocalization to or from the cytosol in response to a drug (Figure 2A). The analysis is based on extraction of cytosolic proteins using digitonin for selective permeabilization of the plasma membrane. Relative quantification of proteins in the cytosol and the remaining fraction (non-cytosol, membrane-enriched) between untreated and FTI-treated cells can then reveal treatment-induced relocalizations (Figure 2B). Such relocalizations can be identified as both FTI-dependent decreases in the non-cytosolic fraction and/or increases in the cytosolic fraction (as shown by western blot for HRAS; Figure 2C). The quantitative readout of the relocalizations depends on (1) the baseline distribution of the protein between the subcellular compartments in the untreated cells; (2) protein turnover; and (3) extent of target farnesylation. The in-depth proteomics analysis of all generated fractions across the four CLs resulted in the identification and quantification of 12,676 proteins (9,982–10,769 proteins per CL). As an estimation of protein relocalization, for each protein, we calculated a delta score (ΔReLoc) that considers both the increase in the cytosolic fraction and the decrease in the non-cytosolic fraction (Figure 2D; Table S5). We then applied a set of criteria for relocalizing proteins: (1) log_2_ ΔReLoc >0.4, (2) opposite directions of change in the two fractions, and (3) DEqMS adj. *p* value <0.05 in at least one of the fractions.

Overall, this analysis indicated a widespread impact of the FTI treatment on protein localization, with significant relocalization from non-cytosolic compartment to the cytosol of 35–182 proteins per CL (Figures 2E and S2). In total, 412 proteins were indicated in this analysis, including 61 proteins with a CXXX motif. Out of the relocalizing proteins with a CXXX motif, only 25 (41%) were identified at all in our prenylation pull-down experiment, with 14 meeting the criteria as PFP in at least one CL. This indicates that the pull-down experiments have limited sensitivity. Many proteins without a farnesylation motif were also identified as relocalizing, which are likely secondary effects due to, for example, protein interactions or intracellular signaling. Nonetheless, overlapping the PFPs and FTI-induced relocalization analysis allowed the identification of 14 relocalizing farnesylated proteins.

Matching the data for the individual CLs highlighted a list of top 12 farnesylated proteins with significant spatial redistribution after the FTI treatment (Figure 2F). Interestingly, this list included HRAS and NRAS but not KRAS. All three of the Ras family proteins were found to be prenylated (in two, three, and four CLs, respectively) and commonly localized to the secretory neighborhood. However, only HRAS was found to be relocalizing upon the FTI treatment in all four CLs and NRAS only in NCI-H1975 cells (Figures S3A–S3C). These findings suggest that HRAS localization is specifically dependent on farnesylation, which is consistent with previous findings that, unlike HRAS, both KRAS and NRAS can alternatively be geranylgeranylated upon FTI treatment and thus remain membrane-localized.^9^ Observed NRAS relocalization in NCI-H1975 cells indicates that farnesylation and the effect of FTIs may be context-dependent. To investigate this question further, we evaluated the protein levels of the different prenylation enzyme components in NCI-H1975, NCI-H1568, and NCI-H2009 CLs. FNTB encoding the beta subunit of FTase was found to be high in NCI-H1975 and significantly lower in the other two CLs. In contrast, PGGTB1 encoding the beta subunit of geranylgeranyltransferase type I (GGTase-I) was low in NCI-H1975 and significantly higher in the other two CLs (Figure 2G). Both enzymes share the same alpha subunit, FNTA, and there were no significant differences in its protein levels between the CLs. This supports the finding that NRAS may be more reliant on farnesylation than geranylgeranylation in NCI-H1975 cells than in the other two CLs. We investigated the role of FNTB levels further and found that among NRAS-mutant solid tumor CLs, response to tipifarnib correlates with high expression of FNTB (Figure 2H). Further, evaluation of FNTB levels across different cancer CL types indicated that skin/melanoma cells commonly express high levels of FNTB (Figure S9F). As melanoma is also commonly NRAS mutated (15%–20%),^30^ this finding indicates a potential future stratification possibility for investigation of tipifarnib response in melanoma.

The inositol-metabolizing phosphatase INPP5A was one of the two proteins identified as farnesylated and relocalizing after FTI in all four investigated CLs (Figure S3D). INPP5A was recently identified as a drug target in uveal melanoma (UVM) with activating mutations in the guanosine nucleotide-binding protein Q gene (GNAQ) or its paralog guanosine nucleotide-binding protein alpha-11 gene (GNA11), which occur in approximately 90% of these cancers.^31^ Mutations in GNAQ/GNA11 result in overactivation of phospholipase C beta (PLCβ) that produces two cellularly active second messengers, IP3 and diacylglycerol (DAG). Studies have shown that silencing or knockout of INPP5A *in vitro* in GNAQ or GNA11-mutant UVM cells resulted in accumulation of IP3/IP4, hyperactivation of IP3-receptor signaling, increased cytosolic calcium levels, and p53-dependent apoptosis.^31^ *In vivo* experiments further showed that inhibition of INPP5A dramatically reduced tumor burden and metastasis in a mouse model of GNAQ or GNA11-mutant UVM.^31^ Supporting this, evaluation of INPP5A essentiality based on CRISPR knockout data indicated significantly lower cell fitness scores in GNAQ- or GNA11-mutant UVM CLs (Figure S4A).

To evaluate if INPP5A relocalizes after tipifarnib treatment also in UVM cells, we treated GNA11-mutant MP41 cells with 1 μM tipifarnib for 24 h. Cell fractionation of the cells after tipifarnib treatment and harvest showed reduced INPP5A level in the non-cytosolic fraction while INPP5A was below the detection limit in cytosolic fractions, indicating relocalization of INPP5A also in UVM cells (Figure S4B). Further, we evaluated apoptosis in MP41 cells, the GNAQ-mutant UVM CL MP46, and in NSCLC NCI-H2009 cells (Figures S4C and S4D) after tipifarnib (5 μM, 72 h) and lonafarnib (5 μM, 72 h) treatment using flow cytometry. This analysis indicated a significant increase in early apoptotic cells in both UVM CLs after tipifarnib or lonafarnib treatment, whereas no increase was detected in NCI-H2009 cells. We further evaluated apoptosis upon tipifarnib treatment in UVM CLs MP41 and MP46 using western blot. We observed a dose-dependent increase in cleaved PARP, confirming the results from the flow cytometry analysis (Figure S4E). Our data thus indicate that tipifarnib could be evaluated as a potential strategy for inhibition of INPP5A in UVM with GNAQ or GNA11 mutations.

To complete the analysis of FTI-dependent relocalization, we also performed the reciprocal analysis to identify proteins moving from the cytosol to the non-cytosol compartments after tipifarnib treatment (Figure 2I). Strikingly, only one protein, AIFM2, relocalized from the cytosol to the non-cytosol in all four CLs (Figure S3E). AIFM2 encodes FSP1 (ferroptosis suppressor protein 1, from here on referred to as AIFM2) that was recently identified as a key protein in one of the two independent cellular defense mechanisms against ferroptosis, a form of regulated cell death caused by peroxidation of lipids,^32^^,^^33^ which will be discussed further below.

Our findings confirm that, besides the Ras family proteins, FTIs affect and drive relocalization of various substrates, including those relevant for cancer. Consequently, the end cellular outcome of FTI treatment of cancer cells depends on the combined impact of multiple independent farnesylated proteins on multiple cellular processes. For a holistic view of FTIs’ impact on cellular processes, we continued with proteomics profiling of cells after tipifarnib treatment.

### Proteome-wide evaluation of tipifarnib effects on cellular signaling

To further evaluate the impact of FTI treatment on cellular processes, we analyzed the global proteomics data generated in the four CLs after 24-h tipifarnib treatment compared to the controls (Figure 1A). This analysis included the identification and quantification of 11,905 proteins (9,156–10,115 proteins per CL; Figure S5A; Table S6). Differential protein abundance analysis between tipifarnib-treated and control cells indicated widespread changes in all CLs, including proteins across all cellular compartments (Figures S5B and S5D). More pronounced and similar molecular responses to tipifarnib were found in NCI-H1568, NCI-H1975, and NCI-H2009 as compared to NCI-H727 (Figure S5C). This can potentially be explained by the fact that NCI-H727 is of neuroendocrine lineage with high levels of NCAM1 and NRCAM markers, which, according to our public domain data analysis below, may predict lack of response to tipifarnib (Figures 4C and S9A). For the three CLs with a similar response, we further evaluated the impact of tipifarnib at a later time point (48-h treatment) using global proteomics (Figure S5E; Table S6). Furthermore, we performed a time course experiment profiling the response to tipifarnib (6-, 12-, 24-, and 48-h treatments) using global proteomics applied to an additional lung adenocarcinoma CL, NCI-H1944 (Figure S5F; Table S6).

**Figure 3:**
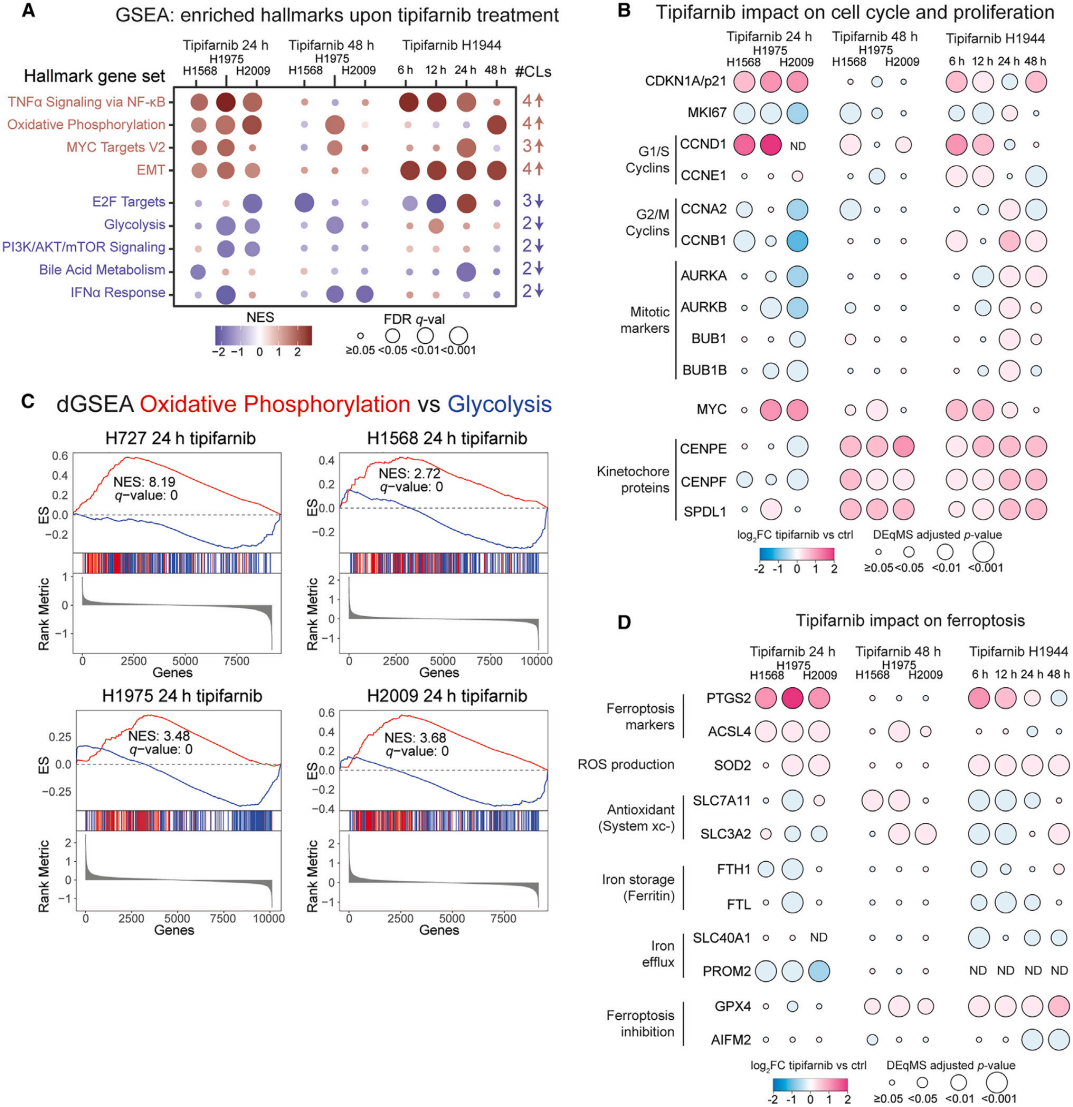
MS-based proteomics reveals that tipifarnib treatment leads to a complex cellular response (A) Gene set enrichment analysis (GSEA) using the hallmarks gene sets. The commonly regulated gene sets are displayed with the corresponding normalized enrichment score (NES) and false discovery rate (FDR) *q* value (q-val). CLs, cell lines; EMT, epithelial mesenchymal transition. (B) Cell cycle and proliferation-related protein levels upon tipifarnib treatment. Fold change (FC) and adjusted *p* values (adj. *p*-val) were calculated using the DEqMS method. (C) Differential GSEA (dGSEA) for oxidative phosphorylation and glycolysis hallmark gene sets. ES, enrichment score; NES, normalized enrichment score. The false discovery rate (FDR) *q* value is displayed. (D) Protein levels of ferroptosis-related proteins. Fold change (FC) and adjusted *p* values (adj. *p*-val) were calculated using the DEqMS method. In all panels, the NCI- prefix has been omitted in the cell line names.

For a broad phenotypic evaluation of FTI effects, we performed gene set enrichment analysis (GSEA) using the “hallmarks” gene sets^34^ and all the global proteomics data generated in the five CLs (Figure S6). This analysis supported the observed deviating impact of tipifarnib on cellular processes in NCI-H727 cells 24 h after treatment. Consequently, we excluded this CL from further analysis related to generalizable treatment effects. The common top up- and downregulated proteins between the remaining CLs are indicated in Figure S7. Based on the GSEA, we focused on the cellular processes that were commonly affected in the remaining four CLs, also considering the normalized enrichment score (NES) and the fraction of leading-edge proteins that were gene-set specific (Figure 3A). Overall, this analysis indicated increases in tumor necrosis factor alpha (TNF-α)/nuclear factor (NF)-κB signaling (4 CLs), oxidative phosphorylation (4 CLs), MYC signaling (3 CLs), and epithelial to mesenchymal transition (EMT, 4 CLs). Further, the GSEA indicated decreases in E2F targets (3 CLs), glycolysis (2 CLs), PI3K/AKT/mTOR signaling (2 CLs), bile acid metabolism (2 CLs), and interferon (IFN) alpha response (2 CLs). Interestingly, the impact of tipifarnib was transient for most processes, except for the increased oxidative phosphorylation and decreased IFN signaling that was still evident in more than one CL 48 h after the tipifarnib treatment. All the highlighted processes were further examined by leading edge analysis, proteomics data evaluation, and literature research and connected back to the farnesylation and subcellular (re)localization results. The selected relevant findings will be discussed later.

The downregulation of the E2F targets gene set indicates that tipifarnib treatment reduces proliferation. FTI treatment has previously been shown to cause a p53-dependent upregulation of p21 (CDKN1A) followed by G1 cell-cycle arrest.^35^ Importantly, the same study also showed that, in the absence of p53/p21, FTIs still reduced cell growth and resulted in mitotic abnormalities and polyploidy. Our proteomics profiling supports these findings with increased levels of CDKN1A and G1/S cyclins and reduced levels of G2/M cyclins, mitotic markers, and a proliferation marker MKI67 in tipifarnib-treated cells as compared to the control (Figure 3B). The largest impact on these proteins was seen in NCI-H2009, which is the most sensitive CL according to the GDSC dataset. Most of these effects were transient, indicating a release from the G1 arrest at later timepoints after the tipifarnib treatment. This release could in part be driven by MYC as previously described^36^ and indicated by the GSEA and increased MYC levels in all four CLs (Figures 3A and 3B). At later time points, we instead detected increased levels of three kinetochore proteins, CENPE, CENPF, and SPDL1, in all four CLs (Figure 3B). Interestingly, these three proteins are known farnesylation substrates, previously shown to be important for the kinetochore attachment to spindle microtubule that drive chromosome segregation during mitosis.^37^ Further, FTI treatment was shown to cause impaired recruitment of all three proteins to kinetochores, resulting in mitotic abnormalities.^37^ Farnesylation of CENPE and CENPF has also been shown to be important for their APC/C-dependent ubiquitination and proteasomal degradation at the end of anaphase to allow normal progression through mitosis.^38^ Our analysis thus supports previous findings showing that inhibition of farnesylation affects proliferation and cell cycle at multiple different levels.

Several metabolism-related gene sets were also commonly regulated upon tipifarnib treatment of the cells. Cancer cells are known to rely on glycolysis instead of oxidative phosphorylation even in the presence of oxygen to allow for anabolism and to control cellular reactive oxygen species (ROS) levels, a phenomenon termed aerobic glycolysis or the Warburg effect.^39^ The simultaneous increase in oxidative phosphorylation and decrease in glycolysis indicated by the GSEA suggest that tipifarnib treatment alters metabolism. To examine the impact of tipifarnib on oxidative phosphorylation and glycolysis in relation to each other, we applied differential GSEA (DGSEA), as this method has been shown to be more predictive of the metabolic state of cancer CLs than standard GSEA.^40^ The DGSEA strongly supported a switch from glycolysis to oxidative phosphorylation in NCI-H727, NCI-H1568, NCI-H1975, and NCI-H2009 cells 24 h after tipifarnib treatment (Figure 3C) and at 48 h in NCI-H1975 cells (Figure S8A). The trend was also observed at 48 h in NCI-H1944 cells; however, it did not reach significance (Figure S8A). This metabolic switch could potentially be linked to reduced PI3K/AKT/mTOR signaling indicated by the GSEA in NCI-H1975 and NCI-H2009 cells as mTOR is a driver of the Warburg effect.^41^ Previous studies have indicated the mTOR-activating protein RHEB as a central downstream target of FTI treatment that results in inhibition of RHEB farnesylation and reduced mTOR signaling.^11^^,^^42^^,^^43^ RHEB is a known substrate of FTase, and like HRAS, the localization of RHEB is exclusively dependent on farnesylation.^2^ In our analysis, RHEB was found farnesylated in all four investigated CLs (Figure 1B). Further, our relocalization analysis suggested either increased cytosolic or decreased non-cytosolic localization of RHEB in NCI-H727, NCI-H1975, and NCI-H2009 after tipifarnib treatment (adj. *p* value: 0.01, 1.0 × 10^−5^, 0.07, respectively, Figure S8B). Interestingly, the smallest impact on metabolism was seen in NCI-H1568 and NCI-H1944 that are both STK11-inactivated, which results in decreased AMPK activation and increased mTOR signaling, potentially protecting the cells from this effect of tipifarnib.

The observed altered metabolism can also be linked to ferroptosis, a form of regulated cell death caused by peroxidation of lipids due to high levels of ROS and intracellular iron. FTIs have previously been shown to induce ROS peaking at 6 h in cancer cells.^44^ Oxidative phosphorylation generates ROS including superoxide (O_2_^−^) that is converted to hydrogen peroxide (H_2_O_2_) by superoxide dismutase 1/2 (SOD1/2) in mitochondria. H_2_O_2_ then diffuses into the cytosol, and in the presence of free iron, H_2_O_2_ can be converted to hydroxyl radical (^−^OH) through the Fenton reaction, resulting in peroxidation of lipids including poly-unsaturated fatty acids (PUFAs). Excessive PUFA peroxidation results in loss of membrane integrity and ferroptosis.^39^ Reduced glycolysis can also directly reduce resistance to ferroptosis as it was recently shown that lactate produced during glycolysis protects cells from ferroptosis.^45^ Based on our findings of altered metabolism as well as AIFM2 relocalization to membranes after tipifarnib treatment, we investigated ferroptosis further using the generated proteomics data (Figure 3D). Supporting the induction of ferroptosis, both PTGS2 (a bona fide marker for lipid peroxidation occurring during GPX4-regulated ferroptosis^46^) and ACSL4 (activates PUFAs for incorporation into membrane-localized lipids^47^) were upregulated after tipifarnib treatment. Further, SOD2 levels were increased and system xc^–^ (SLC7A11 and SLC3A2, cysteine/glutamate antiporter that imports cysteine for conversion into the antioxidant glutathione^48^) were decreased, suggesting increased oxidative stress after tipifarnib treatment. In addition, proteins important for both iron storage through ferritin (FTH1 and FTL) and iron efflux through SLC40A1 and PROM2 decreased after tipifarnib treatment, suggesting increased cellular levels of free iron.^48^ Finally, at later time points we noted a strong increase in the glutathione peroxidase GPX4 that together with AIFM2 protects cells against ferroptosis.^32^ Although no overall increase in AIFM2 was evident after tipifarnib treatment, our subcellular localization analysis clearly showed relocalization of AIFM2 from cytosol to membrane compartments, indicating activation after tipifarnib treatment. Importantly, most of the evidence of ferroptosis after tipifarnib treatment was transient, suggesting that the actions of GPX4 and AIFM2 efficiently protected the cells from ferroptosis-induced death. For a broader insight into the cellular processes affected at the earliest time point after the tipifarnib treatment, we performed GSEA using REACTOME gene sets and the proteomics data generated in NCI-H1944 cells 6 h after the tipifarnib treatment. Interestingly, the top downregulated gene set was related to solute carrier (SLC)-mediated transmembrane transport (Figure S8C), indicating that inhibition of farnesylation results in an altered membrane composition. Many of the cellular systems that regulate ferroptosis localize to membranes and are dependent on different SLCs for proper function. Farnesylation inhibitors could thus interfere with these systems on a general level.

In summary, our analysis of the phenotypic impact of tipifarnib highlights the complexity of the cellular response to farnesylation inhibition. Furthermore, the analysis suggests a potential sequence of events where inhibition of farnesylation results in membrane reorganization with impact on both cellular oxidative stress and free iron levels triggering ferroptosis. It is thus of interest to explore the use of ferroptosis inducers together with FTIs (see later discussion).

### Role of farnesylated proteins and FTIs in lung cancer based on public domain data

**Figure 4:**
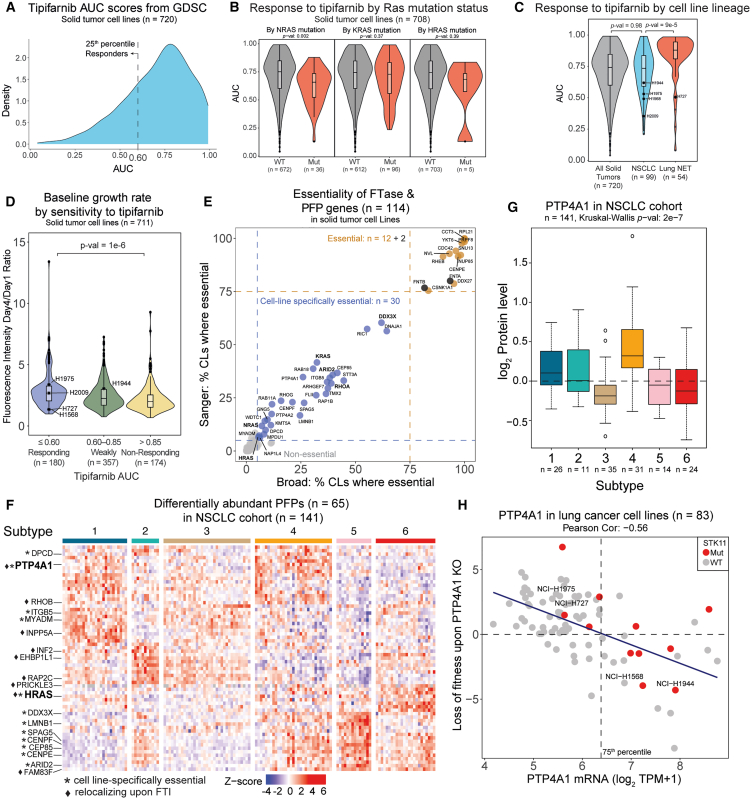
Publicly available data highlight relevance of multiple PFPs in cancer (A) Tipifarnib AUC scores (GDSC1 dataset) in solid tumor cell lines (*n* = 720). (B) Tipifarnib AUC scores by NRAS, KRAS, and HRAS mutation status (WT, wildtype; mut, mutant) per Sanger Institute’s Cell Model Passport annotations in solid tumor cell lines (*n* = 708). (C) Tipifarnib AUC by cell line primary disease (Broad Institute’s DepMap annotation). NSCLC, non-small cell lung cancer; NET, neuroendocrine tumor. *p* value was calculated using Welch’s t test; the size of the groups is indicated in the figure. (D) Cell line growth rate (Sanger Institute’s Cell Model Passport data) by tipifarnib AUC score in solid tumor cell lines (*n* = 711). (E) Essentiality of prenylation candidates (*n* = 114, see Figure 1D) and farnesyltransferase (FTase) components FNTA and FNTB in Broad Institute’s and Sanger Institute’s datasets (*n* = 578 and *n* = 305 solid tumor cell lines [CLs], respectively). Essentiality was defined as a scaled loss of fitness score <0 upon CRISPR-based gene knockout. (F) Heatmap displaying PFPs (see Figure 1D) that were found to be differentially abundant in the non-small cell lung cancer (NSCLC) proteome subtypes published by Lehtiö et al. (2021) (*n* = 65 proteins, *n* = 141 tumors). Differential protein abundance was defined as DEqMS adjusted *p* value <0.01 and absolute log_2_ ratio >0.5 in at least one pairwise comparison between the subtypes. Cell-line-specifically essential proteins as per panel (E) and proteins relocalizing upon farnesyltransferase inhibition (FTI) as per Figure 2E are indicated. (G) PTP4A1 protein levels in the Lehtiö et al. (2021) NSCLC cohort. *p* value was calculated using Kruskal-Wallis test, and the number of samples per subtype is indicated. (H) PTP4A1 mRNA levels (CCLE 21Q4 release) versus loss of fitness score upon PTP4A1 knockout (KO) in lung cancer cell lines (*n* = 83), colored by STK11 mutation status (WT, wildtype; mut, mutant, Sanger Institute’s Cell Model Passport annotation). The corresponding Pearson correlation coefficient (Pearson Cor.) and a linear regression are displayed. For panels (B–D), *p* values (*p*-val) were calculated using Welch’s two-sided t test. In panels (C and D), the NCI- prefix has been omitted in the cell line names.

FTIs were initially developed as targeted therapies for Ras oncogene-driven cancers. However, the initial clinical trials did not show efficacy.^2^ As shown earlier, FTI treatment has widespread effects on cellular processes via multiple farnesylation target proteins, hence it is likely that alternative cancer driving events can expose cell vulnerability to FTIs. To identify potentially predictive markers of response to tipifarnib, we used the GDSC drug sensitivity dataset with area under the curve (AUC) as the measure of sensitivity (a low AUC indicates response to the drug; Figure 4A). We first explored the role of Ras mutations. It was previously reported that, unlike HRAS, KRAS and NRAS can escape FTI-induced prenylation inhibition via alternative geranylgeranylation.^9^ Therefore, we expected that HRAS mutations would be more predictive of response to tipifarnib than KRAS or NRAS mutations. Interestingly, however, neither KRAS nor HRAS mutations predicted response to tipifarnib in solid tumor CLs. In case of HRAS, the lack of statistical significance could be explained by the small group size for HRAS-mutant CLs (*n* = 5). On the other hand, there was a significant difference in tipifarnib AUC between NRAS-mutant and wild-type CLs (*p* value = 0.002) (Figure 4B). This is supported by our relocalization profiling that found NRAS relocalizing upon tipifarnib treatment in one CL (Figure S3). This finding reinforces that farnesylation and FTI effects are context dependent. Therefore, we next looked at response depending on the CL lineage. We found that there was no difference in tipifarnib response between NSCLC and all solid tumor CLs. But lung neuroendocrine tumor (NET) CLs (94% of which belong to the small cell lung cancer subtype) were more resistant than NSCLC (*p* value = 8.95 × 10^−5^) (Figure 4C). It should be noted that within NSCLC, there are large-cell lung carcinomas that are also of neuroendocrine lineage. Therefore, we also evaluated response by neuronal lineage marker levels NCAM1 and NRCAM (defined as mRNA expression above or below the median) and, indeed, cells with high marker levels were more resistant to tipifarnib (*p* value = 2.41 × 10^−5^) (Figure S9A). Lung NET CLs have distinct growth rates, compared to NSCLC CLs (Figure S9B); therefore, we investigated the relationship between CL growth rate and response to tipifarnib across all solid tumor CLs. We found that CLs that are sensitive to tipifarnib (AUC <0.6) had significantly higher growth rates than non-responding CLs (AUC >0.85, *p* value = 8.12 × 10^−7^) (Figure 4D). It should be noted that despite statistical differences in AUC between the described groups, there is still a wide range of response within groups, and there is a need for predictive biomarkers.

Tipifarnib’s target, FTase, has numerous substrates beyond Ras family proteins that may also be important or even essential for FTI efficacy. To further investigate the relevance of the identified PFPs as targets of cancer therapy, we used publicly available omics datasets and resources. FTase proteins (FNTA and FNTB) as well as eight of the 116 PFPs are known cancer-relevant proteins as per COSMIC Cancer Gene Census^49^ or TCGA^50^ (KRAS, HRAS, NRAS, RHEB, RHOA, DDX3X, ARID2, and SAV1). In addition, CRISPR essentiality data^51^ revealed that 12 PFPs are essential in >75% of solid tumor CLs, and further 30 PFPs are essential in 5%–75% of CLs, thus highlighting their importance for general cell fitness (Figure 4E). We then evaluated the expression of PFPs in our previously published resource characterizing proteomic subtypes of NSCLC.^52^ The analysis revealed 65 differentially abundant PFPs across the six subtypes (Figure 4F). We chose to focus on the PFPs that are both cell-line-specifically essential and are differentially abundant in lung cancer subtypes (*n* = 11 proteins), as we believe they may reveal biology that could guide patient stratification for FTI therapy selection. Further, we cross-analyzed the 11 candidates with our farnesylation-dependent relocalization analysis and found that only two proteins relocalized from the non-cytosolic to the cytosolic fraction upon tipifarnib treatment: HRAS and PTP4A1. We chose to explore the role of PTP4A1 further as it is less investigated than HRAS. Prior to further investigations, we verified the ability to predict gene essentiality based on mRNA expression. We found that, indeed, PTP4A1 levels correlate with loss of fitness score upon PTP4A1 knockout in lung cancer CLs in Broad Institute’s CCLE^53^ and CRISPR Project Score^51^ datasets (Figure 4H). Thus, when evaluating transcriptomics or proteomics data from clinical cohorts, we can infer importance of PTP4A1 in tumors with high PTP4A1 expression. Among the NSCLC proteome subtypes,^52^ PTP4A1 was overexpressed in subtype 4 (Figure 4G). Subtype 4 was previously described by us as immune-cold, metabolically rewired, and enriched for STK11 and KEAP1 mutations and high expression of liver-specific proteins^52^ (Figure S9C). We confirmed the negative correlation of PTP4A1 and STK11 at mRNA level in TCGA data and significantly higher levels of PTP4A1 in STK11mut tumors and CLs (Figures S9D and S9E). In the original publication, subtype 4 NSCLC was studied using a model CL NCI-H1944, which corresponded to the subtype 4 characteristics. Thus, for further exploration into the role of PTP4A1, NCI-H1944 was determined to also be a relevant model, as it expresses high levels of and is dependent on PTP4A1 (Figure 4H).

### PTP4A1 is overexpressed in STK11-inactivated subtype 4 NSCLC and is involved in regulation of tonic IFN signaling

**Figure 5:**
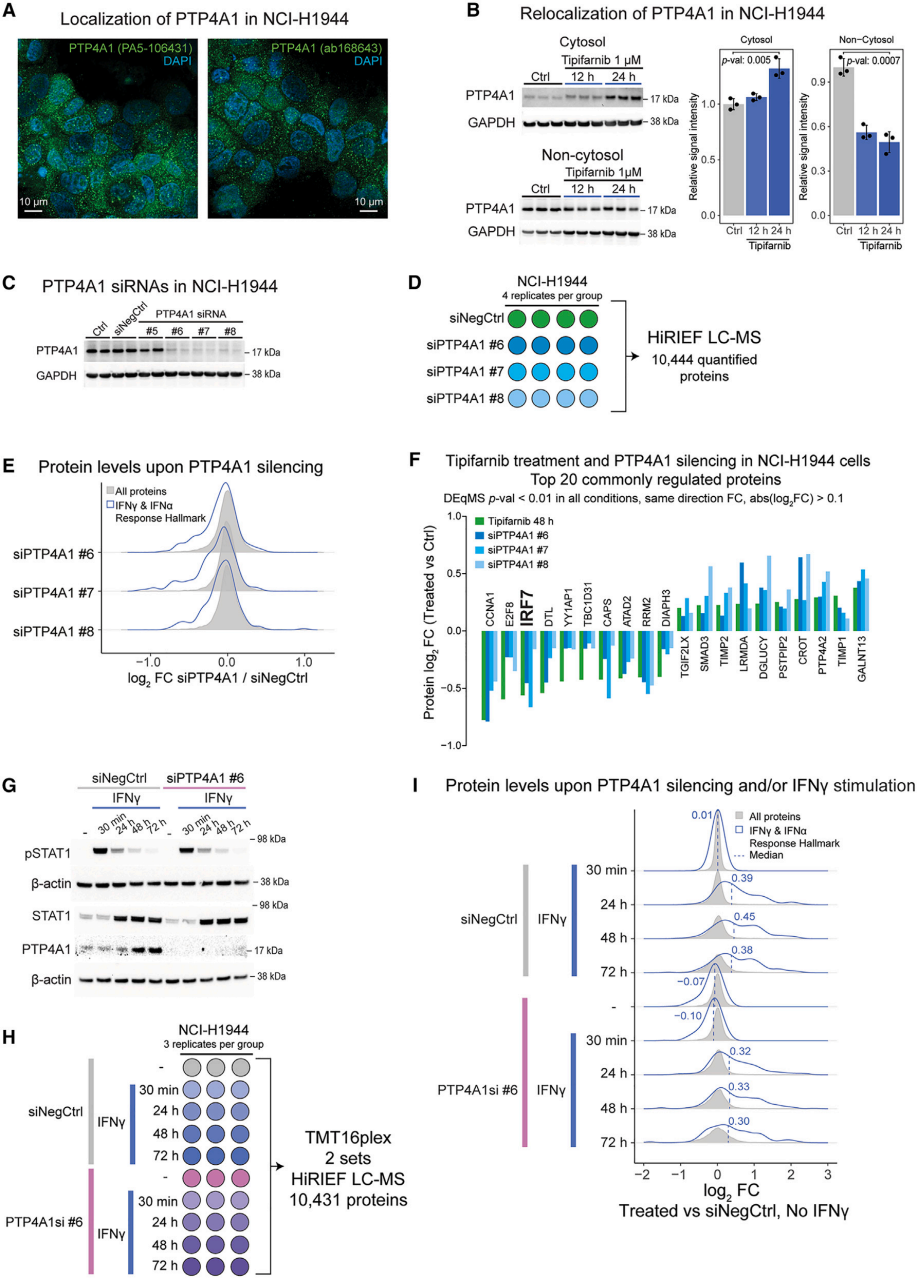
Proteomics analysis implicates PTP4A1 in regulation of tonic IFN signaling (A) Immunofluorescence microscopy evaluating PTP4A1 localization in NCI-H1944 using two different antibodies. (B) Western blot and densitometry analysis of PTP4A1 in cytosolic and non-cytosolic fractions with and without tipifarnib treatment (1 μM, 24 h) in NCI-H1944 cells. Densitometric values were normalized to GAPDH and then the control mean and are represented as mean ± SD (*n* = 3 independent cell cultures). *p* values (*p*-val) were calculated using Welch’s two-sided t test. (C) Western blot analysis of PTP4A1 upon PTP4A1 silencing using four different siRNAs and negative control siRNA (siNegCtrl), *n* = 4 independent cell cultures were performed, and one randomly selected replicate was used for the western blot analysis. GAPDH is shown as the loading control. (D) Experimental design for MS-based evaluation of PTP4A1 silencing effects on the proteome. HiRIEF-LC-MS, high-resolution isoelectric focusing-liquid chromatography-mass spectrometry. (E) Density plots showing fold changes (FC) in protein levels upon PTP4A1 silencing using different siRNAs (*n* = 10,431 proteins, including *n* = 158 proteins in IFN-γ and IFN-α response hallmark gene sets, *n* = 4 independent cell cultures per group). (F) Top 20 proteins commonly regulated by tipifarnib treatment (see Figure 3) and PTP4A1 silencing in NCI-H1944 cells. Fold change (FC) and adjusted *p* values (DEqMS *p*-val) were calculated using the DEqMS method. (G) Western blot analysis of STAT1, pSTAT1, and PTP4A1 upon PTP4A1 silencing and/or IFN-γ treatment (10 ng/mL), *n* = 3 independent cell cultures were performed, and one randomly selected replicate was used for the western blot analysis. β-actin is shown as the loading control. (H) Experimental design for MS-based evaluation of PTP4A1 silencing and IFN-γ treatment effects on the proteome. (I) Density plots showing fold changes (FC) in protein levels upon PTP4A1 silencing and/or IFN-γ treatment (10 ng/mL) (*n* = 8,644 proteins, including *n* = 150 proteins in IFN-γ and IFN-α response hallmark gene sets, *n* = 3 independent cell cultures per group).

As described earlier, our analysis indicated that tyrosine phosphatase PTP4A1 (also called PRL1, phosphatase of regenerating liver 1) may have an oncogenic role in STK11-inactivated NSCLC and that PTP4A1-dependent effects of FTIs in these cancers should be further evaluated. First, we evaluated the subcellular localization of PTP4A1 in our selected model, NCI-H1944, using fluorescence microscopy with two different antibodies against PTP4A1 indicating a vesicular/endoplasmic localization of PTP4A1 in agreement with previous reports^54^ (Figure 5A). Next, we verified farnesylation-dependent relocalization of PTP4A1 by treating cells with tipifarnib followed by cell fractionation and western blot analysis (Figure 5B). To investigate the general role of PTP4A1 in NCI-H1944 cells, we silenced PTP4A1 using four small interfering RNAs (siRNAs) and performed in-depth proteome-wide profiling using the three most effective siRNAs (#6, #7, and #8; Figures 5C and 5D; Table S7). GSEA based on the quantified proteins (*n* = 10,444) indicated that PTP4A1 silencing strongly reduced IFN-α and IFN-γ signaling in all three tested siRNAs (Figures 5E, S10A, and S10B). Furthermore, all three PTP4A1 siRNAs downregulated three proliferation-related hallmark gene sets, namely E2F targets, G2/M checkpoint, and MYC targets, thus supporting NCI-H1944 cells’ dependence on PTP4A1 for fitness and proliferation.

To identify PTP4A1-dependent effects of FTIs at the protein level, we investigated common effects of PTP4A1 silencing and tipifarnib treatment at 48 h in NCI-H1944 cells (Figure 5F). Interestingly, one of the top downregulated proteins common between tipifarnib treatment and PTP4A1 silencing was IRF7, a master regulator of IFN-α/-β transcription,^55^ thus indicating that PTP4A1 may promote IFN signaling. The downregulation of IRF7 after tipifarnib treatment agrees with a previous study indicating that tipifarnib treatment in combination with oncolytic M1 virus infection reduces IRF7 expression and increases virus replication *in vitro* and *in vivo.*^56^ A potential molecular explanation for the decreased IRF7 levels after tipifarnib treatment or PTP4A1 silencing is through upregulation of FOXO3. FOXO3 negatively regulates IRF7 transcription and is itself negatively regulated by Akt phosphorylation, resulting in relocalization of FOXO3 from the nucleus to cytoplasm and degradation of FOXO3.^57^ By limiting the transcription of IRF7, FOXO3 thus prevents leakiness in IFN signaling in the absence of viral infection. In our experiments, tipifarnib treatment increased FOXO3 levels as seen in NCI-H1568 and NCI-H1944 cells (Figure S10C) or increased nuclear localization as indicated by a strong increase in non-cytoplasmic FOXO3 in NCI-H1975 (the CL with the strongest downregulation of IRF7 levels and IFN-α hallmark gene set 24 h after tipifarnib) (Figure S10E). FOXO3 was also upregulated in PTP4A1-silenced cells for two of the three siRNAs (Figure S10B).

Next, we investigated the impact of PTP4A1 silencing on IFN response by treating NCI-H1944 cells with IFN-γ. In both PTP4A1-silenced and control cells, IFN-γ treatment clearly increased STAT1 phosphorylation 30 min after treatment, followed by an increase in STAT1 protein levels (Figure 5G). Interestingly, IFN-γ treatment also increased PTP4A1 levels at 48 and 72 h post-stimulation (Figure 5G), suggesting that PTP4A1 could be involved in feedback signaling. Proteomic profiling of the IFN-γ response (Figure 5H; Table S7) again indicated reduced IFN signaling in PTP4A1-silenced untreated cells but that IFN-γ treatment efficiently upregulated IFN-stimulated genes (ISGs; Figure 5I). Under homeostatic conditions (i.e., without infection or inflammation), low-level constitutive “tonic” IFN-signaling enables cells to mount an effective and rapid response to IFNs produced upon infection or inflammation. Tonic IFN signaling has been shown to induce transcription of specific subsets of ISGs that are dependent on IFNAR1 in homeostatic conditions including IFIT1-3, MX1/2, OAS genes, IRF9, STAT1, and STAT2.^58^ Interestingly, PTP4A1 silencing showed a stronger impact on ISGs associated with tonic/homeostatic IFN signaling, whereas classical IFN-γ responsive ISGs were less affected (Figure S10B).

Tonic IFN signaling is driven by vanishingly low quantities of type I IFNs (IFN-α/-β) via type I IFN receptor (IFNAR), which in turn results in baseline transcription of IRF9, STAT1, and STAT2. These three transcription factors can then form the ISGF3 complex. Under homeostatic conditions, unphosphorylated ISGF3^59^ or STAT2/IRF9^60^ complexes bind to genes with IFN-stimulated response elements (ISREs) and drive basal expression of ISGs. Importantly, our proteomics analysis shows that both PTP4A1 silencing and tipifarnib reduced levels of the three members of ISGF3 and various other ISGs (Figures S10B–S10D).

The impact of PTP4A1 silencing on tonic IFN signaling could potentially be explained by reduced levels of both IRF7, as discussed earlier, and DDX58 (RIG-I), a pattern recognition receptor that senses microbial or self-nucleic acids and triggers IRF7 activation and type I IFN gene transcription^61^ (Figure S10B). Supporting our findings, PTP4A1 has previously been shown to protect RIG-I from SHP2/PTPN11-dependent degradation, and knock-out of PTP4A1 reduced RIG-I levels and abolished RIG-I-dependent upregulation of IFNB1 in HepG2 hepatocellular carcinoma cells.^62^ Importantly, RIG-I has previously been shown to enhance the IFN-α response in the absence of infection by amplifying IFN-α effector signaling via strengthening STAT1 activation in both *in vitro* and *in vivo* models of hepatocellular carcinoma.^63^

Our data provide compelling evidence that tipifarnib treatment reduces tonic IFN signaling via PTP4A1 and that the mechanism involves upstream signaling components including RIG-I and IRF7. Nonetheless, the data must be interpreted with caution due to extensive feedback signaling within the pathway. Both IRF7 and IRF9 are also ISGs themselves and transcribed by ISGF3, resulting in positive feedback and amplification of type I IFN signaling.^64^ Our own analysis also clearly demonstrates this as IFN-γ treatment results in upregulation of all ISGF3 components, modulates the levels of IFNAR1, and upregulates FOXO3 at later time points to name a few examples (Figure S10B). Our data showing upregulation of PTP4A1 48 h after IFN-γ treatment suggests that PTP4A1 could be part of IFN feedback signaling through impact on IRF7 or RIG-I levels or even at the receptor level as we see increasing IFNAR1 levels 72 h after IFN treatment that are largely missing in the PTP4A1-silenced cells (Figure S10B). Future targeted studies are needed to reveal the exact mechanism of PTP4A1 effects on IFN signaling, the potential connection to cell fitness, and the clinical value of these findings in relation to cancer and inflammation.

### Tipifarnib triggers ferroptosis in lung cancer CLs

**Figure 6:**
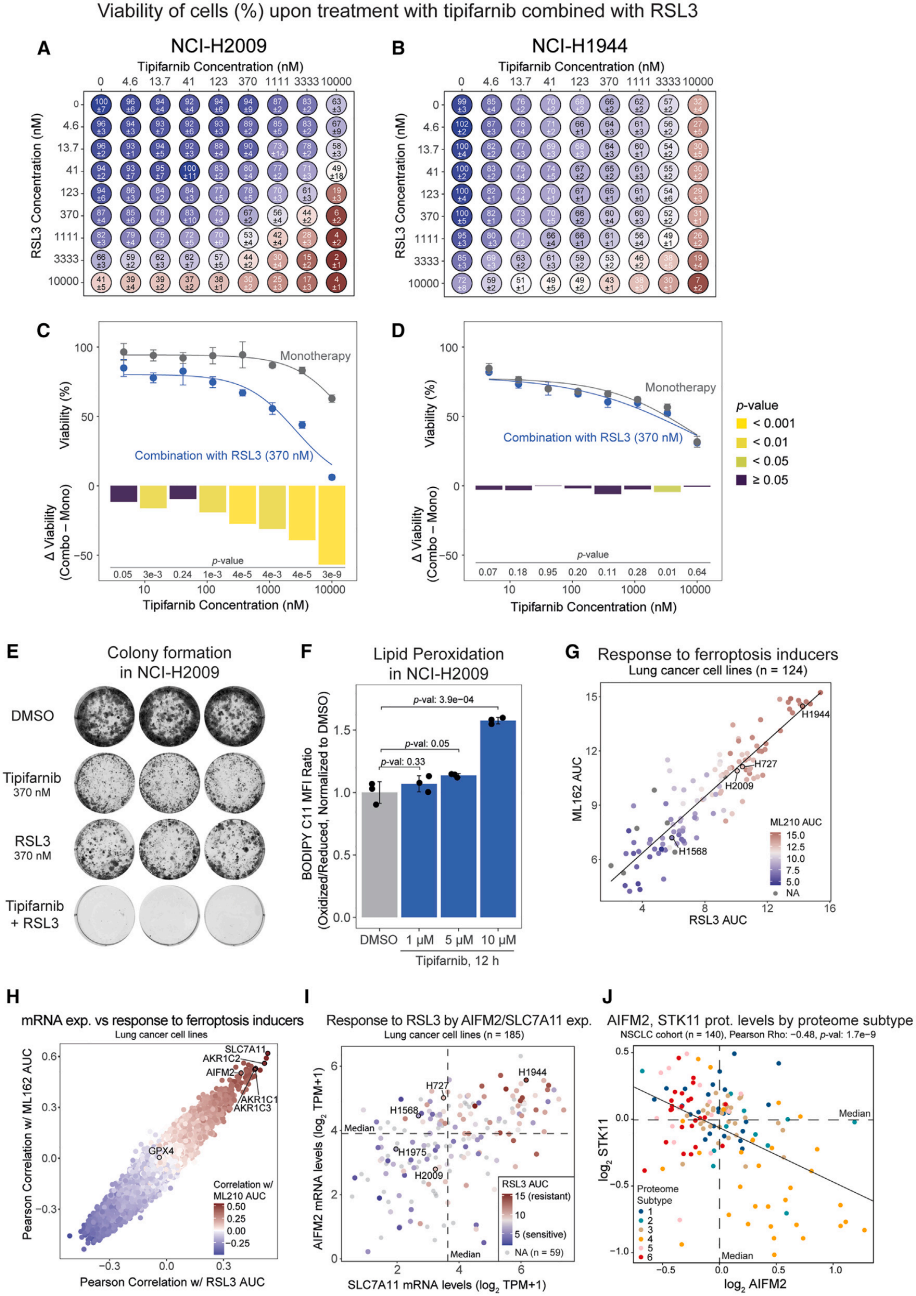
Tipifarnib triggers ferroptosis and is synergistic with a ferroptosis inducer RSL3 in a subset of cell lines (A and B) Viability of cells upon tipifarnib and/or ferroptosis inducer RSL3 treatment in NCI-H2009 and NCI-H1944 cells, evaluated in a 72-h CellTiter-Glo-based assay in a 384-well plate format. The values presented are mean ± SD (*n* = 8 wells for drug monotherapies and *n* = 3 wells for drug combinations). (C and D) Drug response curves in NCI-H2009 and NCI-H1944 cells derived from the data presented in panels (A and B), regression lines derived using nls() function in R. The differences between the viability upon monotherapy (Mono) and drug combination (Combo) treatment were calculated, and the two-sided t test *p* values (*p*-val) are presented. Also see Figures S11A–S11F. (E) Colony formation in NCI-H2009 cells treated with tipifarnib and RSL3 alone or in combination for 11 days. *n* = 3 wells were performed, all are shown. Also see Figures S11G and S11H. (F) Lipid peroxidation in NCI-H2009 cells measured as the ratio of oxidized to reduced BODIPY C11 autofluorescence-corrected median fluorescence intensity (MFI). The values were normalized to the DMSO control and presented as mean ± SD (*n* = 3 independent cell cultures). Also see Figures S11I and S11J. (G) Ferroptosis inducers (RSL3, ML162, ML210) AUC scores in lung cancer cell lines (*n* = 124) published in Bersuker et al. (2019). (H) Pearson correlation of mRNA levels (CCLE 21Q4 release, *n* = 19177 gene symbols) versus ferroptosis inducer AUC were calculated for lung cancer cell lines (*n* = 126). The correlation coefficient was plotted for RSL3 and ML162, colored by ML210. (I) mRNA levels of AIFM2 and SLC7A11 (CCLE 21Q4 release) in lung cancer cell lines (*n* = 185), colored by RSL3 AUC. (J) Protein levels of AIFM2 and STK11 in NSCLC cohort published by Lehtiö et al. (2021), colored by proteome subtype (*n* = 140 tumors). In panels (G) and (I), the NCI- prefix has been omitted in the cell line names.

Our relocalization analysis as well as the global proteomics analysis discussed earlier indicated ferroptosis activation upon FTI treatment of lung cancer CLs. Therefore, we hypothesized that a combination of tipifarnib with drugs targeting ferroptosis defense systems such as a GPX4 inhibitor (GPX4i) RSL3 could be synergistic. To test this hypothesis, we measured sensitivity of all five lung cancer CLs to tipifarnib and RSL3 alone and in combination by a viability assay 72 h post-treatment across nine concentrations for each drug (0–10 μM). The highest tested concentration of tipifarnib or RSL3 monotherapy resulted in 32%–63% and 18%–72% viability across the five CLs, respectively (Figures 6A, 6B, and S11A–S11C). Strikingly, the tipifarnib/RSL3 combination resulted in almost complete killing of the cells (2%–7% viability) in all CLs, except NCI-H727 (20% viability). We next calculated the synergy score using the Loewe model and found the FTI/GPX4i combination to be synergistic in NCI-H2009, NCI-H1568, and NCI-H1975 cells with synergy scores of 14%, 11%, and 6%, respectively. However, the combination was not synergistic in NCI-H727 or NCI-H1944 cells (synergy scores −3% and 1.4%, respectively). The synergy differences are also indicated by tipifarnib dose-response curves with and without 370 nM RSL3 (Figures 6C–6D and S11D–S11F). As previously discussed, NCI-H727 are of neuroendocrine lineage and do not respond to tipifarnib in the same manner as the rest of the CLs potentially explaining the lack of synergy in these cells.

To validate the synergistic effect of combined tipifarnib and RSL3 treatment, we performed a colony formation assay in the three CLs where synergy was detected in the short-term assay. Treatment of NCI-H2009 cells (Figure 6E) with a combination of tipifarnib and RSL3 (370 nM, each) as well as NCI-H1568 and NCI-H1975 (100 nM tipifarnib/370 nM RSL3; Figures S11G and S11H) resulted in complete cell killing. In all three CLs, the corresponding monotherapies failed to efficiently kill the cancer cells. To confirm our hypothesis that tipifarnib treatment triggers ferroptosis, explaining the synergistic effect shown earlier, we assayed lipid peroxidation after tipifarnib using flow cytometry and the lipid peroxidation sensor BODIPY C11. In both NCI-H2009 cells (Figures 6F and S11J) as well as in NCI-H1568 cells (Figures S11I and S11J), i.e., the two CLs with the strongest synergistic effect, a dose-dependent increase in lipid peroxidation was evident already at 12 h after the tipifarnib treatment.

To further investigate the differences in response to the tipifarnib and RSL3 combination treatment in the different CLs, we used recently published drug sensitivity data where several GPX4 inhibitors were tested in cancer CLs.^32^ Response to the GPX4 inhibitors RSL3, ML162, and ML210 (represented as AUC) highly correlated across the investigated lung cancer CLs (Figure 6G). The data indicated NCI-H1944 as one of the most resistant CLs, which agrees with our experimental data for RSL3 monotherapy (Figures 6A, 6B, and S11A–S11C). Next, we performed a correlation analysis to find genes with mRNA expression that correlates with response to the GPX4 inhibitors (Figure 6H). SLC7A11 and AIFM2 expression correlated with GPX4 inhibitors’ AUC (i.e., resistance to RSL3, ML162, and ML210) in lung cancer CLs, in agreement with the previously reported correlation in non-hematopoietic CLs in general.^32^ Our analysis also indicated that the three members of the aldo-keto-reductase-1C (AKR1C) family (AKR1C1, AKR1C2, AKR1C3) correlated with resistance to GPX4 inhibitors in lung cancer CLs (Figure 6H). AIFM2,^65^ SLC7A11,^66^ and AKR1C family^67^ are all regulated by NRF2, a transcription factor with overactivation in STK11mut/KEAP1mut lung cancer cells, which explains the high mRNA levels of these genes in NCI-H1944 that harbors STK11 and KEAP1 mutations (Figures 6I and S12A). AIFM2 and SLC7A11 have well-known roles in protection against ferroptosis as has been discussed earlier. Further, inhibition of the AKR1C family proteins increased sensitivity to ferroptosis-inducing agents, indicating that AKR1C also provides protection against ferroptosis.^67^ Levels of GPX4 itself did not, however, correlate with response to the GPX4 inhibitors. The ferroptosis-protective role of GPX4 is dependent on its ability to use glutathione (GSH) to detoxify lipid hydroperoxides into lipid alcohol, and SLC7A11 plays a role in this process by transporting extracellular cystine for GSH biosynthesis. Based on metabolomics data previously generated for the CCLE CLs,^68^ SLC7A11 correlates with glutathione levels in lung cancer CLs (Figure S12B). Further, using the same metabolomics resource, we investigated the levels of triacylglycerols (TAGs) with different numbers of double bonds as an estimation of cellular PUFA levels (Figures S12C–S12E). This analysis indicated that glutathione levels negatively correlate with PUFA levels, thus supporting the role of glutathione and SLC7A11 in detoxification of lipid peroxides (Figure S12B). Together this analysis suggests that the lack of response to the tipifarnib/RSL3 combination treatment in NCI-H1944 cells could be explained by the STK11 and KEAP1 mutations resulting in upregulation of AIFM2, SLC7A11, and AKR1C and the resultant resistance to ferroptosis. Importantly, AIFM2 protein levels are also high in NSCLC proteomic subtype 4 that is associated with STK11 and KEAP1 mutations (Figures 6J and S12F). Further confirming the dependence of AIFM2 expression on STK11 and KEAP1 mutations, significantly higher levels of AIFM2 at both protein and mRNA levels were seen in lung cancer samples with STK11/KEAP1 mutations (Figures S12G and S12H). In summary, our findings suggest a potential value of FTI/GPX4i combination therapy in NSCLC. Furthermore, we suggest stratification strategies for future development, where NSCLC subtype 4 classification, STK11/KEAP1 mutations, or neuroendocrine lineage would be negative predictive factors.

## Discussion

Protein farnesylation is known to impact subcellular localization of substrate proteins through membrane anchoring, which allows them to interact with other proteins and enables spatially resolved signaling. Farnesyltransferase inhibitors (FTIs) such as tipifarnib and lonafarnib are approved or currently investigated in clinical trials in the context of cancer and other diseases, yet a systems-level knowledge of the impact of these drugs on hundreds of potential farnesylation substrates and on the proteome in general is largely missing. In this study, we present a proteome-wide investigation of the subcellular localization of prenylated/farnesylated proteins and the impact of tipifarnib on protein localization and cellular signaling at the protein level. Our functional proteomics investigation indicates several potentially translatable findings.

With evidence at multiple levels, our analysis indicates that tipifarnib treatment induces ferroptosis, highlighting potential synergy between FTIs and compounds targeting ferroptosis defense mechanisms in lung cancer cells. Even though there are currently no clinically approved ferroptosis-inducing drugs, this field is rapidly evolving with multiple preclinical compounds already developed, targeting different mechanisms of the ferroptosis process.^69^ As with other targeted therapies, the efficiency of ferroptosis inducers is likely to be context-dependent and potentially stronger in a combination therapy setting. Our data here indicate that treatment with FTIs in combination with ferroptosis-targeting compounds is synergistic and should be evaluated further. Our analysis also provides indications that such combination therapy would be less effective in STK11-/KEAP1-mutated lung cancer and lung cancer of neuroendocrine lineage. The reason for this stems from the differences in both response to FTIs and differences in ferroptosis defense systems where STK11/KEAP1-mutant lung cancer harbors very effective anti-ferroptosis systems through SLC7A11/GPX4, AIFM2, and AKR1C. Our analysis also highlights a potential mechanism for the synergy as tipifarnib treatment alters cell metabolism and results in a switch from glycolysis to oxidative phosphorylation. This switch is likely to result in increased ROS production,^70^ which could contribute to increased lipid peroxidation and ferroptosis. Further, both our relocalization analysis and our global proteomics profiling indicate that inhibition of farnesylation results in general changes the architecture of membranes, with potential impact on both ROS detoxification and membrane integrity.

Our analysis also indicates that the impact of FTIs on specific substrates is dependent on the cellular balance between FTase and GGTase-I. Of specific interest in this context is the dependence of tipifarnib response on FNTB levels in NRAS mutant cells. Specifically, melanoma cells have relatively high FNTB levels and are commonly mutated for NRAS.^30^ Tipifarnib in combination with sorafenib has been investigated in a phase II clinical trial of untreated metastatic melanoma. Although the combination was well tolerated, it did not show sufficient activity over standard of care, and the authors indicated lack of molecularly guided treatment stratification as a limitation of the study.^71^ Evaluation of FNTB levels as well as NRAS mutation status could provide potentially treatment-predictive information that could guide the design of future clinical trials including tipifarnib or other FTIs in melanoma.

INPP5A was one of two proteins identified as farnesylated and relocalizing after FTI in all four CLs examined. Importantly, INPP5A was recently shown to be a highly specific vulnerability in UVM with activating mutations in GNAQ/GNA11, which occur in 90% of these cancers.^31^ This study highlighted INPP5A as a target of cancer therapy in UVM. Currently, there are no available inhibitors of INPP5A, and it has in general proven difficult to develop clinically useful phosphatase inhibitors. Our finding here indicates that FTIs should be further investigated as an alternative approach to inhibit INPP5A in UVM.

Intriguingly, our targeted investigation indicates that tipifarnib treatment or silencing of the farnesylation substrate PTP4A1 in STK11-mutant lung cancer cells results in inhibition of proliferation as well as impaired tonic interferon signaling. Cancer-intrinsic low-level interferon signaling provides a cell fitness benefit and treatment resistance in many cancer types.^72^ Whether the negative impact on proliferation after PTP4A1 silencing in STK11-mutant cells is a consequence of inhibited interferon signaling or not remains to be further studied. PTP4A1 is overexpressed in STK11-mutant NSCLC, but the functional impact of PTP4A1 in lung cancer is so far unknown. STK11 inactivation in lung cancer is associated with an immune cold phenotype with limited infiltration of immune cells^52^ and resistance to immune checkpoint inhibitors.^73^ Given the central role of interferon signaling in the interplay between the immune system and cancer cells, it is tempting to speculate that overexpression of PTP4A1 could affect anti-cancer immune responses. In fact, it was recently shown that STK11-mutant lung cancer is associated with interferon gamma signaling in tumors with low infiltration of CD8^+^ T cells. The authors further speculate that this finding could indicate that STK11 inactivation may contribute to reduced immune infiltration in patients with activated interferon gamma signaling.^74^ Our data thus indicate that the impact of tipifarnib treatment in patients may alter the tumor-immune system interactions with possible implications in immunotherapy.

Our analysis supports previous findings that FTI treatment results in a mitotic block through reduced farnesylation of spindle proteins CENPE/CENPF/SPDL1. The impact on spindle proteins is a general effect seen in all our investigated CLs. Our public domain analysis further indicates that CENPE is essential in almost all CLs, consistent with its central role in mitosis. Inhibition of CENPE function through FTI treatment could thus mimic the effects of microtubular poisons, commonly used anticancer agents, and cause mitotic arrest and mitotic catastrophe.^75^ This effect of FTIs is likely stronger in rapidly proliferating cells, which may explain the association between response to FTIs and growth rate in short-term viability assays used in drug screening. It is unclear if this effect of FTIs is transient, if it is synergistic to other anti-cancer therapies, and if it is specifically effective in certain cancer types. Of potential interest in this context, CENPE has been suggested as a target for cancer therapy in high-grade brain tumors as a less toxic alternative to microtubule poisons that are effective but cause severe side effects.^76^

Our functional proteomics investigation of protein farnesylation, protein localization, FTI-dependent relocalization, and global impact of FTIs on the proteome provides a valuable resource for further studies of signaling in cancer and biology in general. Our study presents a combined analysis of farnesylation, protein localization, and response to farnesyl transferase inhibition. The resource includes the following: (1) metabolic label pull-down data in four CLs covering 5,888 proteins including 607 proteins containing CXXX motif indicating 116 prenylated/farnesylated proteins (PFPs); (2) SubCellBarCode data in four CLs with subcellular localization covering 13,434 proteins; (3) FTI-induced relocalization data in four CLs covering 12,676 proteins including 1,104 relocalizing proteins (in either direction) and 61 proteins with CXXX motif relocalizing from the non-cytosolic to cytosolic compartments; (4) global profiling of FTI effects on the proteome in five CLs covering 13,345 proteins. To meet requirements of FAIR Data Principles,^77^ all generated data are easily accessible and visualized through Supplementary Tables and R scripts. Lastly, we here demonstrate a new method for simplified protein relocalization analysis that can be easily and robustly performed to investigate the impact of treatment on protein localization.

In summary, our study highlights the fact that inhibition of farnesylation results in a highly complex and context-dependent cellular response. A more thorough understanding of the molecular effects of FTI treatment can reveal hereto unknown dependencies and potential new strategies for patient stratification and combination therapy. The data presented here will hopefully contribute such understanding to future studies.

### Limitations of the study

It should be noted that we do not provide direct evidence of prenylation/farnesylation since we do not detect the probe-tagged peptides directly. Instead, we infer protein prenylation by identification of non-modified peptides that are mapping to proteins with statistically significant enrichment in the metabolic pull-down experiments. The focus of the study is the effects of FTI treatment that targets farnesylation; however, the probe for the pull-down experiment performed in this study is not specific to farnesylation and may result in the identification of geranylgeranylated proteins as well. For this reason, the indicated proteins are referred to as prenylated/farnesylated proteins (PTPs). We overcome these uncertainties by complementing the data with protein relocalization analysis upon tipifarnib treatment, which facilitates narrowing down the candidates to farnesylated proteins. In the current study, we did not assess the direct role of the CXXX motif for PTP4A1 localization and activity. Instead, we focused on the role of PTP4A1, assuming that its farnesylation is necessary for its activity. This study is based on CLs and public domain data; however, no validation experiments have been performed *in vivo*.

## Resource availability

### Lead contact

Further information and requests for resources and reagents should be directed to and will be fulfilled by the lead contact, Lukas M. Orre (lukas.orre@ki.se).

### Materials availability

This study did not generate new unique reagents.

### Data and code availability

• Proteomics data have been deposited at ProteomeXchange via PRIDE and are publicly available as of the date of publication. Accession numbers are listed in the key resources table. Original western blot images and flow cytometry data have been deposited on Mendeley and are publicly available as of the date of publication. The DOI is listed in the key resources table. Microscopy data reported in this paper will be shared by the lead contact upon request. This paper analyses several publicly available datasets; the information for these is provided in the key resources table.
• The code for mining the human proteome sequences to generate a list of proteins containing a CXXX motif is deposited on Zenodo. The DOI is listed in the key resources table. The code to generate a “prenylated/farnesylated protein passport” is provided as supplemental information.
• Any additional information required to reanalyze the data reported in this work paper is available from the lead contact upon request.

## Acknowledgments

The confocal microscopy imaging was carried out at the Biomedicum Imaging Core Facility (BIC) at Karolinska Institutet. L.M.O. received funding from 10.13039/501100007232Radiumhemmets Forskningsfonder (projects 184133 and 214133), the 10.13039/501100002794Swedish Cancer Society (22 2000 Pj 01 H), and Karolinska Institutet (2019-00905). Y.P. was funded by Vetenskapsrådet (2022-03517). We acknowledge the Biomedicum Flow Cytometry core facility (Karolinska Institutet), supported by K.I., for providing cell analysis services, technical expertise, and scientific input.

## Author contributions

The project was conceptualized by L.M.O., J.L., and Y.P. and supervised by L.M.O. Prenylation/farnesylation profiling, subcellular localization, and relocalization experiments were performed by Y.P. Validation experiments were performed by Y.P., O.B., and S.M. High-throughput drug combination testing was performed by O.B., G.G., and B.S.-L. MS analysis was performed by Y.P. and G.M. Downstream bioinformatics analysis was performed by O.B., Y.P., and L.M.O. CXXX motif search and bioinformatics consultation was done by T.A. Sequence enrichment in the CXXX motif was performed by A.D.W. Visualizations were prepared by L.M.O. and O.B. The manuscript was written by L.M.O. and O.B. All authors contributed to finalizing the manuscript and approving the final version.

## Declaration of interests

The authors declare no competing interests.

## STAR★Methods

### Key resources table

**Table:** 

REAGENT or RESOURCE	SOURCE	IDENTIFIER
**Antibodies**		

Rabbit anti-PRL1/PTP4A1	Thermo Fisher Scientific	Cat# PA5-106431; RRID: AB_2854103
Rabbit anti-INPP5A	Thermo Fisher Scientific	Cat# PA5-45906; RRID: AB_2607840
Mouse anti-PRL1/PTP4A1	Abcam	Cat# ab168643; RRID: AB_3111273
Goat anti-rabbit IgG H&L (HRP)	Abcam	Cat# ab205718; RRID: AB_2819160
Goat anti-rabbit Alexa Fluor 488	Abcam	Cat# ab150077; RRID: AB_2630356
Goat anti-mouse Alexa Fluor 488	Abcam	Cat# ab150113; RRID: AB_2576208
Mouse anti-H-Ras	Sigma-Aldrich	Cat# MAB3291; RRID: AB_94790
Mouse anti-GAPDH	Sigma-Aldrich	Cat# G8795; RRID: AB_1078991
Mouse anti-β-actin	Santa Cruz Biotechnology	Cat# sc-47778; RRID: AB_626632
Goat anti-mouse IgG H&L (HRP)	GE Healthcare	Cat# NA931V
Rabbit anti-ATP2A2/SERCA2 (D51B11)	Cell Signaling Technology	Cat# 9580; RRID: AB_10827913
Rabbit anti-cleaved PARP (Asp214)	Cell Signaling Technology	Cat# 9541; RRID: AB_331426

**Chemicals, peptides, and recombinant proteins**		

Ammonium bicarbonate	Sigma-Aldrich	Cat# A6141
Biotin-PEG4-alkyne	Sigma-Aldrich	Cat# 764213
BODIPY 581/591 C11	Thermo Fisher Scientific	Cat# D3861
Bovine serum albumin (BSA) protein standard	Sigma-Aldrich	Cat# A7030
Chloroform	Sigma-Aldrich	Cat# C2432
Clarity Western ECL substrate	Bio-Rad	Cat# 1705061
Copper(II) sulfate (CuSO4)	Sigma-Aldrich	Cat# 209198
DAPI	Thermo Fisher Scientific	Cat# 00-4959-52
Digitonin	Sigma-Aldrich	Cat# D141
Dimethyl sulfoxide (DMSO)	Sigma-Aldrich	Cat# D2650
Dithiothreitol (DTT)	Sigma-Aldrich	Cat# 43815
Dynabead MyOne Streptavidin C1 resin	Invitrogen	Cat# 65001
Ethylenediaminetetraacetic acid (EDTA)	Thermo Fisher Scientific	Cat# 1860851
Farnesyl alcohol azide (F-azide-OH)	MedChemExpress	Cat# HY-N0504
Fetal bovine serum (FBS)	Sigma-Aldrich	Cat# F7524
Formic acid	Honeywell	Cat# 94318
Halt protease inhibitor cocktail (100x)	Thermo Fisher Scientific	Cat# 78430
4-(2-hydroxyethyl)-827 1-piperazineethanesulfonic acid) (HEPES)	Gibco	Cat# 15630
Iodoacetamide	Sigma-Aldrich	Cat# I1149
Lithium dodecyl sulfate (LDS) sample loading buffer	Invitrogen	Cat# NP0007
Lonafarnib	SelleckChem	Cat# S2797
Lovastatin	Larodan AB	Cat# CAY-13269-1
Magnesium chloride (MgCl2)	Sigma-Aldrich	Cat# M1028
Methanol	Sigma-Aldrich	Cat# 34860
NeutrAvidin Agarose resin	Thermo Fisher Scientific	Cat# 53151
Opti-MEM medium	Thermo Fisher Scientific	Cat# 31985062
Paraformaldehyde	Thermo Fisher Scientific	Cat# J61899.AK
Penicillin-streptomycin (P/S)	Sigma-Aldrich	Cat# P4333
Phosphate-buffered saline (PBS)	Sigma-Aldrich	Cat# D8537
Recombinant human IFNγ	Bio-Techne	Cat# 285-IF-100
RNAiMAX transfection reagent	Invitrogen	Cat# 13778150
(1S,3R)-RSL3 (referred to as RSL3)	Cayman Chemical	Cat# CC-19288
RPMI-1640 cell culture medium	Sigma-Aldrich	Cat# R2405
Sodium chloride (NaCl)	Sigma-Aldrich	Cat# S7653
Sodium dodecyl sulfate (SDS)	Sigma-Aldrich	Cat# 71725
TAMRA-Biotin-Alkyne	Click Chemistry Tools	Cat# 1366-5
TBS with Tween 20 buffer (TBST)	Thermo Fisher Scientific	Cat# 28360
Tris[(1-benzyl-1H-1,2,3- triazol-4-813 yl)methyl]amine (TBTA)	Sigma-Aldrich	Cat# 678937
Tris(2-carboxyethyl)phosphine hydrochloride (TCEP)	TCI Chemicals	Cat# T1656
Tipifarnib	MedChemExpress	Cat# HY-10502
TMT-10plex reagent	Thermo Fisher Scientific	Cat# 90110
TMT-16plex reagent	Thermo Fisher Scientific	Cat# A44520
TMT-18plex reagent	Thermo Fisher Scientific	Cat# A52045
Triton X-100	Active Motif	Cat# 101031
TrypLE	Gibco	Cat# 12604013
Trypsin (MS-grade)	Thermo Fisher Scientific	Cat# 90057
Urea	Sigma-Aldrich	Cat# U1250

**Critical commercial assays**		

DC protein assay	Bio-Rad	Cat# 5000111
CellTiter-Glo 2.0	Promega	Cat# G9243
eBioscience Annexin V Apoptosis Detection Kit	Thermo Fisher Scientific	Cat# 88-8102-72
MycoAlert detection kit	Lonza	Cat# LT07-118
Mycostrip 50	InvivoGen	Cat# rep-mysnc

**Deposited data**		

Proteomics (farnesylated protein pull-down data)	ProteomeXchange	PXD050725
Proteomics (subcellular localization and relocalization data)	ProteomeXchange	PXD050490
Proteomics (global effects of tipifarnib data)	ProteomeXchange	PXD050480
Proteomics (effects of PTP4A1 silencing and/or IFN stimulation data)	ProteomeXchange	PXD050446
Unprocessed western blots	This paper	https://doi.org/10.17632/bfgcnv8ynk.1
Cell line annotations	DepMap portal	https://depmap.org/portal/
Mutation status of cell lines	Sanger Institute’s Cell Model Passports	https://cellmodelpassports.sanger.ac.uk/ (cancer driver mutations, v. 20230202)
Mutation status of uveal melanoma cell lines	DepMap portal	https://depmap.org/portal/ (23Q4 release, GNA11 and GNAQ hotspot mutations)
Gene essentiality data (Sanger v1 and Broad 20Q2 combined)	Pacini et al.^51^	N/A
Gene essentiality data for uveal melanoma cell lines (23Q4+Score Chronos)	DepMap portal	https://depmap.org/portal/ (23Q4 release)
Cell line mRNA expression data	DepMap portal	https://depmap.org/portal/ (CCLE 21Q4 release)
The Pan-Cancer Atlas and lung adenocarcinoma (LUAD) gene expression data	TCGA Research Network	http://cancergenome.nih.gov/
Drug sensitivity data (GDSC1 and GDSC2 screening sets)	Genomics of Drug Sensitivity in Cancer (GDSC) database	https://www.cancerrxgene.org/
Ferroptosis inducers sensitivity data	Bersuker et al.^32^	N/A
Metabolomics cell line data	Li et al.^68^	N/A
NSCLC proteomics data	Lehtiö et al.^52^	N/A
Protein sequences	UniProt	Accession AUP000005640

**Experimental models: Cell lines**		

NCI-H727	ATCC	CRL-5815; RRID: CVCL_1584
NCI-H1568	ATCC	CRL-5876; RRID: CVCL_1476
NCI-H1944	ATCC	CRL-5907; RRID: CVCL_1508
NCI-H1975	ATCC	CRL-5908; RRID: CVCL_1511
NCI-H2009	ATCC	CRL-5911; RRID: CVCL_1514
MP41	ATCC	CRL-3297; RRID: CVCL_4D12
MP46	ATCC	CRL-3298; RRID: CVCL_4D13

**Oligonucleotides**		

Negative control siRNA	Qiagen	Cat# 1027310
PTP4A1 siRNA SI02225874 (Hs_PTP4A1_5)	Qiagen	Part of FlexiTube GeneSolution GS7803 Cat# 1027416
PTP4A1 siRNA SI03024630 (Hs_PTP4A1_6)	Qiagen	Part of FlexiTube GeneSolution GS7803 Cat# 1027416
PTP4A1 siRNA SI03065118 (Hs_PTP4A1_7)	Qiagen	Part of FlexiTube GeneSolution GS7803 Cat# 1027416
PTP4A1 siRNA SI03113180 (Hs_PTP4A1_8)	Qiagen	Part of FlexiTube GeneSolution GS7803 Cat# 1027416

**Software and algorithms**		

ProteoWizard v.3.0.19127	Chambers et al.^78^	RRID: SCR_012056
MS-GF+	Kim et al.^79^	RRID: SCR_015646
Percolator	Käll et al.^80^	RRID: SCR_005040
Nextflow pipeline	Lehtiö lab	https://doi.org/10.5281/zenodo.10940091
OpenMS	Lange et al.^81^	RRID: SCR_012042
R v4.4.1	R Project	RRID: SCR_001905
DEqMS v3.19	Zhu et al.^27^	RRID: SCR_025605
clusterProfiler()	Yu et al.^82^	RRID: SCR_016884
Differential gene set enrichment analysis (DGSEA)	Joly et al.^40^	RRID: SCR_025604
”Prenylation/farnesylation passport” code	This paper	supplemental information
CXXX motif search	This paper	https://doi.org/10.5281/zenodo.13872530
ImageJ	NIH	RRID: SCR_003070
FlowJo	BD Biosciences	RRID: SCR_008520

### Experimental model and study participant details

Lung cancer cell lines, NCI-H727 (ATCC CRL-5815, RRID: CVCL_1584), NCI-H1568 (ATCC CRL-5876, RRID: CVCL_1476), and NCI-H1944 (ATCC CRL-5907, RRID: CVCL_1508), NCI-H1975 (ATCC CRL-5908, RRID: CVCL_1511), NCI-H2009 (ATCC CRL-5911, RRID: CVCL_1514) were purchased from LGC Standards (ATCC). Uveal melanoma cell lines MP41 (ATCC CRL-3297; RRID: CVCL_4D12) and MP46 (ATCC CRL-3298; RRID: CVCL_4D13) were purchased from LGC Standards (ATCC). The cell lines were cultured in RPMI-1640 (Sigma-Aldrich, cat. No. R2405) with 10% fetal bovine serum (FBS, Sigma-Aldrich, cat. No. F7524) and 1% penicillin/streptomycin (P/S, Sigma-Aldrich, cat. No. P4333). All cells were tested using MycoAlert detection kit (Lonza, cat. No. LT07-118) or Mycorstrip detectiom kit (InvivoGen, cat. No. rep-myscnc) and were found to be negative for Mycoplasma contamination.

### Method details

#### Metabolic labeling of proteins

The cells were treated with 25 μM lovastatin overnight to deplete the cellular reserves of farnesyl pyrophosphate (FPP). The cells were incubated further with metabolic labeling reagent (25 μM F-azide-OH/10 μM lovastatin) for 24 h. The cells were then pre-incubated in the standard cell culture medium containing 1 μM tipifarnib or DMSO (control) for 1 h. The medium was then supplemented with 25 μM of F-azide-OH and the cells were incubated for 23 h. The cells were harvested using TrypLE and the proteins were extracting using a lysis buffer (4% SDS, 25 mM HEPES, pH 7.6) without DTT as it would reduce the azide-labeled proteins.

#### Biotin labeling of proteins (click chemistry)

The click reagent mixture was prepared fresh containing 0.5 mM capture reagent (TAMRA-Biotin-Alkyne for in-gel fluorescence and western blot analysis or Biotin-PEG_4_-alkyne for LC-MS/MS analysis), 5 mM TCEP, 0.5 mM TBTA, and 5 mM CuSO_4_. The click reagent mixture was added to the protein lysate and the lysate was incubated on a shaker at room temperature for 1 h. The click reaction was quenched by the addition of EDTA (final concentration 25 mM).

#### Excess biotin-alkyne removal

To remove excess biotin-alkyne, protein precipitation was performed. Methanol (4X volume), chloroform (1X volume), and ultrapure water (3X volume) were sequentially added into the protein lysate after the click reaction, the mixture was vortexed and centrifuged (14,000 × g, 5 min, room temperature). The top layer was removed, leaving the protein layer intact at the phase interface. Methanol (800 μL) was added, the sample was vortexed, and the protein pelleted as before. The supernatant was aspirated, and the protein pellet was again washed in methanol (800 μL) by vortexing and sonication to break the pellet. After the final centrifugation step and removal of the methanol, the pellet was air-dried for 5 min. Proteins were then re-suspended in 2% SDS, and 10 mM EDTA in PBS (as low volume as possible). Once dissolved, the samples were diluted using PBS for a final concentration of 0.2% SDS. The protein concentration was determined by DC Protein Assay with BSA as a standard.

#### Pull-down of F-azide tagged proteins

Even quantities of the protein samples were incubated with Dynabead MyOne Streptavidin C1 resin (20 μL per 100 μg protein, prior to use washed thrice with PBS) for 3 h at room temperature. The beads were washed thrice with 0.2% SDS in PBS (3 × 0.5 mL) and eluted by heating in 1X LDS sample loading buffer (95°C, 5 min).

#### Protein digestion and TMT 10plex labeling

The protein samples were incubated with NeutrAvidin Agarose resin (100 μL per 1 mg protein) for 3–4 h at room temperature and mixing at minimum 900 rpm. The beads were pelleted (3,000 × g, 3 min) and the supernatant was removed. The beads were sequentially washed thrice in 1% SDS in PBS (3 × 0.5 mL), 4 M urea in PBS (2 × 0.5 mL), and 50 mM ammonium bicarbonate (5 × 0.5 mL). For each wash step, the beads were gently vortexed for 1 min, followed by pelleting in a microcentrifuge (3,000 × g, 3 min). After the final wash, the beads were re-suspended in 50 mM ammonium bicarbonate (50 μL). DTT (5 μL, 100 mM in 50 mM ammonium bicarbonate) was added and the beads were incubated at 55°C under shaking for 30 min. The beads were washed twice with 50 mM ammonium bicarbonate (2 × 0.5 mL) by vortexing and pelleting as before, leaving the beads covered with 50 μL supernatant after the second wash. Iodoacetamide (5 μL, 100 mM in 50 mM ammonium bicarbonate) was added and the beads were incubated at room temperature for 30 min in the dark. The beads were washed as before. MS-grade trypsin (5 μL, 0.2 μg/μL in 50 mM ammonium bicarbonate) was added and the beads were incubated under shaking at 37°C overnight. The beads were pelleted, and the supernatant was collected. The beads were washed with 0.1% formic acid in ultrapure water (80 μL) under gentle shaking for 10 min. The beads were pelleted, and the supernatants pooled. The peptide samples were labeled with TMT-10plex reagent according to the standard protocol from the supplier. The labeled samples were pooled, cleaned using strata-X-C columns (Phenomenex, cat. No. 8B-S029-TAK) and dried in a vacuum centrifuge (Electron Savant SpeedVac Concentrator, Thermo Fisher Scientific). The protocol then proceeded with a 4-h gradient LC-MS/MS analysis (see below).

#### Tipifarnib treatment of cells

For the subcellular relocalization analysis, the cells were cultured with medium containing 1 μM tipifarnib for 24 h prior to subcellular fractionation, the controls were left untreated. To evaluate the global effects of tipifarnib at 24 h in NCI-H727, NCI-H1568, NCI-H1975, NCI-H2009 cells, 5% of the cell lysate prepared for the farnesylated protein characterization (see above) was aliquoted for the total proteome analysis. For the global effects evaluation at 48 h in NCI-H1568, NCI-H1975, NCI-H2009 cells, the cells were cultured in three overnight prior to the treatment, after which the standard culture medium was changed to medium containing 1 μM tipifarnib. The controls were left untreated. The cells were then cultured for 48 h before harvest. For the timecourse experiment in NCI-H1944 cells, the cells were cultured overnight prior to the treatment. The standard culture medium was changed to medium containing 1 μM tipifarnib, the cells were then harvested after 6, 12, 24, or 48 h. The controls were cultured for 48 h in medium containing DMSO.

#### Cytosol and non-cytosol fractionation

To separate cells into cytosolic and non-cytosolic subcellular fractions. The cells were seeded the day before the fractionation and grown to 60–70% confluence in four 100-mm plates (four cell culture replicates). The fractionation procedure was adapted from Orre et al. (2019)^28^ and Arslan et al. (2022).^29^ Briefly, the cells were washed with ice-cold PBS. A digitonin solution (42 μg/mL digitonin, 2 mM DTT, 2 mM MgCl_2_, 150 mM NaCl, 200 μM EDTA and 20 mM HEPES, pH 7.6) was added and the cells were incubated for 7 min under gentle rocking at 4°C. The solution was then collected and stored as the cytosolic fraction. The cells were then washed twice with ice-cold PBS and scraped down to obtain a cell pellet as the non-cytosolic fraction. The eight non-cytosolic fractions (four from the control and four from the tipifarnib-treated samples) were lysed by the addition of lysis buffer (4% SDS, 1 mM DTT, and 25 mM HEPES, pH 7.6) followed by heating for 5 min at 95°C and sonication for 1 min. The eight cytosol fractions were untreated. The protein concentration was measured using the DC protein assay with BSA as a standard. The resulting 16 fractions were digested by a modified SP3-based protocol.^83^ The peptide concentration was measured using the DC protein assay. Thereafter, 100 μg of peptides from each digested fraction were labeled with TMTpro 16plex reagent according to the manufacturer’s protocol. The labeled samples were pooled, cleaned using strata-X-C columns (Phenomenex, cat. No. 8B-S029-TAK) and dried in a vacuum centrifuge (Electron Savant SpeedVac Concentrator, Thermo Fisher Scientific). The protocol then proceeded with HiRIEF peptide fractionation (see below).

#### Comprehensive SubCellBarCode characterization

The full version of SubCellBarCode analysis was performed according to the original publications.^28^^,^^29^ Briefly, the cells were seeded the day before fractionation and grown to 70–80% confluency in two 150-mm diameter plates (cell culture replicates). The cells were washed with ice-cold PBS and fractionated into five subcellular fractions: FS1, FS2, FP1, FP2 and FP3. The generated subcellular fractions were then prepared for LC-MS/MS-DDA analysis (see below) with TMT 10plex labeling. The subcellular classifications were generated by the SubCellBarCode pipeline.

#### PTP4A1 silencing and IFNγ treatment

To perform siRNA-based PTP4A1 silencing, the cells were seeded in 6- or 10-cm dishes the day before transfection for an estimated confluence of 50–60% at transfection. The cells were cultured in RPMI-1640 medium with 10% FBS and without an antibiotic. Transfection of siRNA was performed according to the supplier’s protocol. Briefly, RNAiMAX transfection reagent was diluted 17.7-fold in Opti-MEM medium. Each of the PTP4A1 (#6, #7, #8) and negative control siRNAs (10 μM) were diluted 10-fold in Opti-MEM medium. The diluted siRNA and RNAiMAX reagent were mixed (1:1 ratio) and incubated at room temperature for 5 min. The RNA-lipid complexes were then added to the cells for a final siRNA quantity of 56 and 148 pmol in 6- and 10-cm dishes, respectively. The medium was replaced with standard medium with an antibiotic 24 h after transfection. The cells were then cultured for further 48 h before harvesting (total culturing of 72 h after transfection).

For IFNγ stimulation, the medium was replaced with IFNγ-containing medium (10 ng/mL) 48-h after transfection with PTP4A1 siRNA #6. The cells were then cultured for further 30 min, 24, 48 and/or 72 h. The controls were harvested without the addition of IFNγ-containing medium.

#### Proteomics sample preparation

Cells from tipifarnib treatment, PTP4A1 silencing and/or IFNγ stimulation experiments were prepared for in-depth proteomics. The cells were lysed by the addition of lysis buffer (4% SDS, 1 mM DTT, and 25 mM HEPES, pH 7.6) followed by heating for 5 min at 95°C and sonication for 1 min. The protein concentration was measured using the DC protein assay with BSA as a standard. The proteins were digested by a modified SP3-based protocol.^83^ The peptide concentration was measured using the DC protein assay. Thereafter, 100 μg of peptides from each digested fraction were labeled with TMT reagent according to the manufacturer’s protocol. The labeled samples were pooled, cleaned using strata-X-C columns (Phenomenex, cat. No. 8B-S029-TAK) and dried in a vacuum centrifuge (Electron Savant SpeedVac Concentrator, Thermo Fisher Scientific). The protocol then proceeded with HiRIEF peptide fractionation.

#### Sample pre-fractionation using HiRIEF

The TMT-labeled peptides, 300–400 mg, were separated by immobilized pH gradient - isoelectric focusing (IPG-IEF) using the HiRIEF method as described previously^25^ on pH 3–10 strips for all experiments and additionally on pH 3.7–4.9 strips for comprehensive SubCellBarCode characterization, 48-h and timecourse tipifarnib experiments, as well as the three PTP4A1 siRNAs experiment. Peptides were extracted from the strips by a prototype liquid handling robot, supplied by GE Healthcare Bio-Sciences AB. A plastic device with 72 wells was put onto each strip and 50 mL of MilliQ water was added to each well. After 30 min incubation, the liquid was transferred to a 96-well plate and the extraction was repeated 2 more times with 35% acetonitrile (ACN) and 35% ACN, 0.1% formic acid in MilliQ water, respectively. The extracted peptides were dried in Speed-Vac and dissolved in 3% ACN, 0.1% formic acid.

#### MS-DDA-based proteomics for HiRIEF samples

Extracted peptide fractions were separated using an Ultimate 3000 RSLCnano system coupled to a Q Exactive HF or Exploris (Thermo Fischer Scientific, San Jose, CA, USA). Samples were trapped on an Acclaim PepMap nanotrap column (C18, 3 mm, 100 Å, 75 μm × 20 mm, Thermo Scientific), and separated on an Acclaim PepMap RSLC column (C18, 2 μm, 100 Å, 75 μm × 50 cm, Thermo Scientific). Peptides were separated using a gradient of mobile phase A (5% DMSO, 0.1% FA) and B (90% ACN, 5% DMSO, 0.1% FA), ranging from 6% to 37% B in 30–90 min (depending on IPG-IEF fraction complexity) with a flow of 0.25 μL/min. The mass spectrometry was operated in a data-dependent manner, selecting top 5 precursors for fragmentation by HCD. The survey scan was performed at 60,000 resolution from 300 to 1500 m/z, with a max injection time of 100 ms and target of 1 x 10^6^ ions. For generation of HCD fragmentation spectra, a max ion injection time of 100 ms and AGC of 1 x 10^5^ were used before fragmentation at 30% normalized collision energy, 30,000 resolution. Precursors were isolated with a width of 2 m/z and put on the exclusion list for 60 s. Single and unassigned charge states were rejected from precursor selection.

#### MS-DDA-based proteomics for pull-down samples

Q-Exactive online LC-MS analysis was performed using a Dionex UltiMate 3000 RSLCnano System coupled to a Q-Exactive mass spectrometer (Thermo Scientific). From each sample, 3 μL were injected. Samples were trapped on a C18 guard desalting column (Acclaim PepMap 100, 75 μm × 2 cm, nanoViper, C18, 5 μm, 100 Å), and separated on a 50-cm long C18 column (Easy Spray PepMap RSLC, C18, 2 μm, 100 Å, 75 μm × 15 cm). The nano capillary solvent A was 95% water, 5% DMSO, 0.1% formic acid; and solvent B was 5% water, 5% DMSO, 95% acetonitrile, 0.1% formic acid. At a constant flow of 0.25 μL min^−1^, the curved gradient went from 6% solvent B up to 43% solvent B in 180 min, followed by a steep increase to 100% solvent B in 5 min.

FTMS master scans with 60,000 resolution and a mass range of 300–1500 m/z, followed by data-dependent MS/MS (30,000 resolution) on the top 5 ions using higher energy collision dissociation (HCD) at 30% normalized collision energy. Precursors were isolated with a 2 m/z window. Automatic gain control (AGC) targets were 1e6 for MS1 and 1e5 for MS2. Maximum injection times were 100 ms for MS1 and MS2. The entire duty cycle lasted ∼2.5 s. Dynamic exclusion was used with 60 s duration. Precursors with unassigned charge state or charge state 1 were excluded. An underfill ratio of 1% was used.

#### Protein identification and quantification

Peptide and protein identification was performed as described previously in Zhu et al. (2018).^84^ Briefly, Orbitrap raw MS/MS files were converted to mzML format using msConvert from the ProteoWizard tool suite (v.3.0.19127).^85^ Spectra were then searched using MSGF+ (v2017.07.21)^79^ and Percolator (v3.1),^86^ where search results from all HiRIEF fractions of each TMT set were grouped for Percolator target/decoy analysis. All searches were done against the human protein database of Ensembl 91 and 105 in a Nextflow pipeline (https://github.com/lehtiolab/nf-workflows, commit: 898bb20). MSGF+ settings included precursor mass tolerance of 10 ppm, fully tryptic peptides, maximum peptide length of 50 amino acids and a maximum charge of 6. Fixed modifications were TMT-10plex, TMT-16plex or TMT-18plex on lysines and peptide N-termini, and carbamidomethylation on cysteine residues. A variable modification was used for oxidation on methionine residues. Quantification of TMT-10plex, TMT-16plex, or TMT-18plex reporter ions was done using OpenMS project’s IsobaricAnalyzer (v2.0). Peptide spectrum matches (PSM) found at 1% FDR (false discovery rate) were used to infer gene identities.

Protein quantification by TMT-10plex, TMT-16plex, or TMT-18plex reporter ions was calculated using TMT PSM ratios to the reference TMT channels and normalized to the sample median. The median PSM TMT reporter ratio from peptides unique to a gene symbol was used for quantification. Protein FDRs were calculated using the picked-FDR method using gene symbols as protein groups and limited to 1% FDR.^87^

#### In-gel fluorescence and western blot analysis

SDS-PAGE separation was performed according to the standard supplier’s protocol using NuPAGE 4–12% Bis-Tris gels (Invitrogen, Thermo Fisher Scientific), and the detection for TAMRA fluorescence was performed using the iBright CL1500 imaging system (Invitrogen). Western blot analyses were performed according to the standard supplier’s protocol using NuPAGE 4–12% Bis-Tris gels, PVDF iBlot Transfer Stacks (Invitrogen), and iBlot 2 Dry Blotting System (Invitrogen). Anti-H-Ras (1:1000 dilution), anti-GAPDH (1:2000 dilution), anti-PRL1/PTP4A1 (1:600 dilution), anti-K-Ras (1:1000 dilution), anti-INPP5A (1:500 dilution), anti-ATP2A2 (1:500 dilution), and anti-cPARP (1:1000) and anti-β-actin (1:2000 dilution) were diluted in 5% skimmed milk powder in TBST. Clarity Western ECL substrate and iBright CL1500 imaging system (Invitrogen) were used for imaging of the western blots.

#### Apoptosis assay

For the flow cytometry-based evaluation of apoptosis, MP41 and MP46 cell lines were treated with 5 μM tipifarnib and lonafarnib for 72 h with DMSO as control. The cells in the supernatant and the adherent cells harvested using trypsin were pelleted, washed and stained as per manufacturer’s protocol using the eBioscience Annexin V apoptosis Detection Kit. Briefly, samples were stained with Annexin V and 7-AAD dye and were flow cytometrically acquired in BD FACS Canto II (BD Bioscience) in PE and PerCPCy5.5 channels with appropriate compensation matrix applied using single stained tubes. Flow cytometry data was analyzed in Flowjo 10.8.1 software and statistical analysis and data visualizations were performed in R. Thresholds for the quadrants were selected using a visual inspection of the density plots of the PE and PerCPCy5.5 channels signal. Late apoptotic cells were defined as those cells with both PE (Annexin V) and PerCPCy5.5 (7-AAD) signals above the selected thresholds. Early apoptotic cells were defined as those cells that had PE (Annexin V) above the selected threshold but PerCP-Cy5.5 (7-AAD) signal below the selected threshold.

For the western blot-based evaluation of apoptosis, MP41 cells were treated with tipifarnib (5 and 10 μM) for 3 days and MP46 cells (1 and 5 μM) for 5 days, DMSO was used as control. The cells in the supernatant and the adherent cells harvested by scraping were washed in chilled PBS and pelleted, followed by resuspension in a lysis buffer (4% SDS, 25 mM HEPES, 1 mM DTT and 1% Halt protease inhibitor cocktail). The samples were then sonicated for 20 min a bath sonicator and heated for 5 min at 95°C.

#### Confocal microscopy

The cells were grown on an 8-well chamber slide (Thermo Scientific, cat. No. 178599PK) and fixed with 4% paraformaldehyde and permeabilized with 0.25% Triton X-100. Cells were blocked with 2% BSA in 1X PBS for 1 h at room temperature. The cell monolayer was incubated with a primary antibody, anti-PRL1/PTP4A1 (PA5-106431, 1:600 dilution), at the desired concentration for 3 h at room temperature or overnight at 4°C, followed by a secondary antibody goat anti-rabbit Alexa Fluor 488. Nuclei were counterstained with DAPI. Imaging was performed with confocal microscopy (ZEISS LSM 900 with Airyscan 2).

#### Combination drug screen

The drug combination tipifarnib plus RSL3 was tested on NCI-H727, NCI-H1568, NCI-H1944. NCI-H1975, and NCI-H2009 cells using the CellTiter-Glo 2.0 (Promega) assay in a 384-well plate format omitting the edge wells (Corning 3764). The drugs were dispensed into the plates using Echo acoustic liquid handler (Labcyte). The drug plates were then stored until use under an inert atmosphere. Nine concentrations (0 and 4.6 nM–10 μM) were tested for both tipifarnib and RSL3. Each of the drug combinations was tested in triplicates (spread across two 384-well plates), RSL3 and tipifarnib monotherapies were tested with five and eight replicates, respectively. DMSO was added to the monotherapy wells to account for the higher quantity of solvent dispensed into the drug combination wells. DMSO was used as a negative control and BzCl as a positive control with 12 and 10 replicates, respectively. The drugs were dissolved in 5 μL cell culture medium, 20 μL of the cell suspensions were then added using MultiDrop Reagent Dispenser (Thermo Scientific). The seeding density was optimized prior to screening to ensure that each cell line was in the exponential growth phase at 72 h after seeding. Namely, 3000 cells were seeded for NCI-H727, NCI-H1568, and H1944; 1500 cells for NCI-H2009; and 1200 cells for NCI-H1975. After 72-h culturing, CellTiter-Glo 2.0 assay was performed according to the supplier’s instructions. Namely, 30 μL of the reagent were added to each of the wells and after a 10-min incubation at room temperature under shaking in the dark, luminescence was measured using EnSight Plate Reader (PerkinElmer).

Quality control was performed using the Breeze pipeline.^88^ Viability was calculated relative to the negative control wells. The response curves were fitted using the nls() function in R package stats. Synergy scores were calculated using the Loewe model in the SynergyFinger 3.0 tool.^89^

#### Colony formation assay

Cell lines NCI-H2009, NCI-H1568, and NCI-H1975 seeded in six-well plates (Thermo Fisher Scientific, Cat. No. 150628) with 5,000, 10,000, and 500 cells per well, respectively. The cells were then treated with 370 nM RSL3 and/or 100 nM tipifarnib, except for NCI-H2009 where 370 nM tipifarnib was used. The cells were incubated for 11 days with media changes and treatment replenishments every third day. The cells were then fixed using 90% ethanol, air dried briefly and stained with 0.2% crystal violet solution. The colonies were then washed with deionized water and imaged using iBright CL1500 (Thermo Fisher Scientific).

#### Lipid peroxidation assay

Cell lines NCI-H2009 and NCI-H1568 were seeded in 12-well plates (Thermo Fisher Scientific, Cat. No. 150628) and cultured for 16 h, thereafter the cells were treated with 1, 5 or 10 μM tipifarnib or DMSO (control) for 12 h. The cells were then stained with 1.5 μM BODIPY 581/591 C11 for 20 min. The supernatant was collected and centrifuged to pellet the floating cells. The cells in the plate were trypsinized and combined with the cells from the supernatant. The collected cells were washed with PBS and FACS buffer (0.5% BSA in PBS) and finally resuspended in the FACS buffer. Flow cytometric acquisition of samples was performed using FACSCanto II (BD Biosciences) with FITC and PE channels. The data was preprocessed using FlowJo 10.8.1 software, the downstream analysis was performed using R. Autofluorescence for the FITC signal was found to differ between the different tipifarnib concentration samples (determined by Kolmogorov-Smirnov test comparing FITC signal in unstained samples, BH-adjusted *p*-value <0.05), therefore condition-specific autofluorescence correction was applied. On the other hand, autofluorescence for the PE signal was found to be equal between the different conditions (determined by Kolmogorov-Smirnov test comparing PE signal in unstained samples, BH-adjusted *p*-value >0.05 in all pairwise comparisons). Therefore, the mean autofluorescence value was used for the correction. Lipid peroxidation levels were defined as the ratio of oxidized BODIPY C11/reduced BODIPY C11, calculated as the autofluorescence-corrected median FITC signal divided by the autofluorescence-corrected median PE signal.

#### Downstream bioinformatics analysis

All differential protein abundance analyses were performed using the DEqMS method as previously described in Zhu et al. (2019).^27^ The adjusted *p*-value cutoffs used for the analyses are specified in the figures. The resultant fold change values were used to perform gene set enrichment analysis (GSEA) using clusterProfiler() R package and differential GSEA using the DGSEA() R package. Benjamini-Hochberg (BH) method was used to control for multiple hypothesis testing. *Q* value <0.05 was used as the significance cutoff.

#### Analysis of public domain datasets

Cell lines annotations were derived from the Dependency Map (DepMap) portal (https://depmap.org/portal/). Gene essentiality data were taken from Pacini et al. (2021).^51^ Namely, Project score (Sanger v1 and Broad 20Q2 combined) fitness scores as scaled Bayesian factors were used apart from the data for INPP5A presented in Figure S4A. Due to the limited number of uveal melanoma cell lines in the Pacini et al. (2021) dataset, DepMap Public 23Q4+Score (Chronos) data were used. mRNA expression data were downloaded from the DepMap portal (CCLE 21Q4 release). Mutation status of the cell lines was taken from Sanger Institute’s Cell Model Passports (https://cellmodelpassports.sanger.ac.uk/, cancer driver mutations, v. 20230202) apart from the data presented in Figure S4A. Due to the limited number of uveal melanoma cell lines, GNA11 and GNAQ hotspot mutations from the DepMap portal were used instead. Drug sensitivity data (GDSC1 and GDSC2 screening sets) were downloaded from the Genomics of Drug Sensitivity in Cancer (GDSC) database (https://www.cancerrxgene.org/).^24^ The Pan-Cancer Atlas and lung adenocarcinoma (LUAD) gene expression data were derived from the TCGA Research Network (http://cancergenome.nih.gov/). Ferroptosis inducers sensitivity data was downloaded from the supplementary materials of Bersuker et al. (2019)^32^ and the metabolomics cell line data from the supplementary materials of Li et al. (2019).^68^ The NSCLC cohort proteomics data from the supplementary materials of Lehtiö et al. (2021).^52^ To generate a list of proteins containing a CXXX motif at the C-terminal, we used protein sequences from the human proteome (UniProt accession AUP000005640). The code for mining the data is deposited on GitHub (https://github.com/TanerArslan/farnesylation_motif_search/). For the CXXX motif sequence enrichment analysis, *p* values were calculated using Fisher’s exact test. The *p* values were then corrected for multiple hypothesis testing using false discovery rate (FDR). The sequence logo plots were created with ggseqlogo R package.

#### Resource code

The supplementary files contain an R code that can be used to generate a protein’s “prenylation/farnesylation passport” for any protein out of the 15,080 proteins in the dataset. The output PDF file will contain results from the F-azide tagged protein pull-down experiment, subcellular localization and relocalization analyses, as well as protein levels after tipifarnib treatment.

### Quantification and statistical analysis

Details of the statistical analysis can be found in the results, figure legends and methods. Statistical significance was defined as *p* < 0.05 or *p* < 0.01 and specified in text where appropriate.

## Supplemental information

Document S1. Figures S1–S12

Table S1. Summary, related to Figures 1–5

Table S2. Prenylation profiling, related to Figure 1

Table S3. Prenylation candidate support, related to Figure 1

Table S4. Localization, related to Figure 1

Table S5. Relocalization, related to Figure 2

Table S6. Quant after tipifarnib, related to Figure 3

Table S7. Quant PTP4A1si_IFN, related to Figure 5

Data S1. Prenylation/farnesylation passport R code, related to Figures 1–6 Data S2. R data required to run the Data S1 code, related to Figures 1–6.
