# Supplementary material for: Functional-proteomics-based investigation of the cellular response to farnesyltransferase inhibition in lung cancer
Source: iScience. 2025 Jan 21;28(2):111864. doi: 10.1016/j.isci.2025.111864 (PMC11848503; doi:10.1016/j.isci.2025.111864)
Supplement: Document S1. Figures S1–S12 [file mmc1.pdf]

iScience, Volume *28*

Supplemental information

Functional-proteomics-based investigation of the cellular response to farnesyltransferase inhibition in lung cancer

Yanbo Pan, Olena Berkovska, Soumitra Marathe, Georgios Mermelekas, Greta Gudoityte, Amare D. Wolide, Taner Arslan, Brinton Seashore-Ludlow, Janne Lehtiö, and Lukas M. Orre

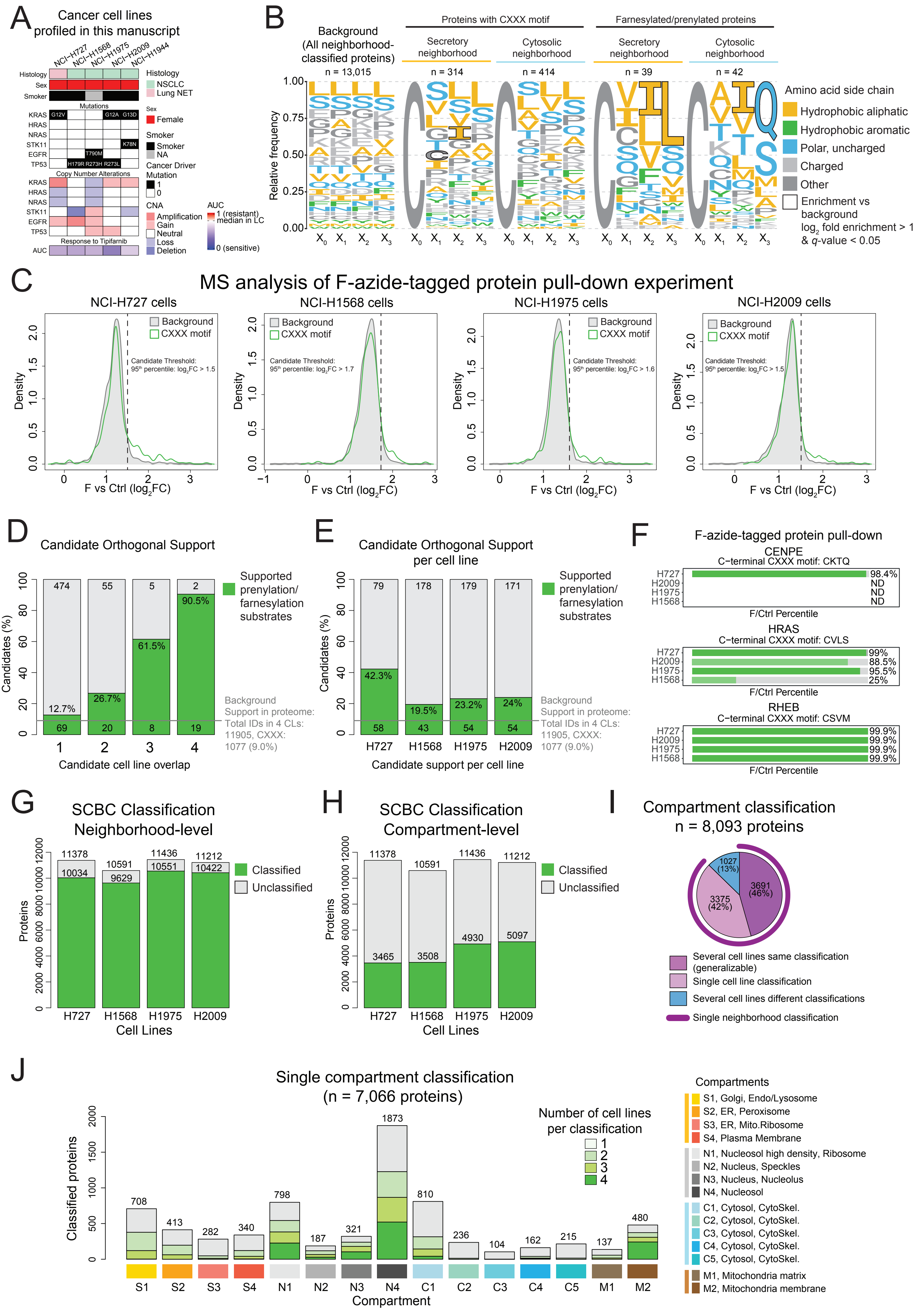
A
Cancer cell lines profiled in this manuscript
B
Background (All neighborhood-classified proteins)
Proteins with CXXX motif
Farnesylated/prenylated proteins
Amino acid side chain
Hydrophobic aliphatic
Hydrophobic aromatic
Polar, uncharged
Charged
Other
C
MS analysis of F-azide-tagged protein pull-down experiment
NCI-H727 cells
NCI-H1568 cells
NCI-H1975 cells
NCI-H2009 cells
D
Candidate Orthogonal Support
E
Candidate Orthogonal Support per cell line
F
F-azide-tagged protein pull-down
G
SCBC Classification Neighborhood-level
H
SCBC Classification Compartment-level
I
Compartment classification n = 8,093 proteins
J
Single compartment classification (n = 7,066 proteins)
Compartments

Figure S1. Identification of prenylated/farnesylated proteins and protein localization profiling in lung cancer cell lines (related to Figure 1).

(A) Cell line annotations from the DepMap portal, Cell Model Passport database and the GDSC dataset. NCI-H727, NCI-H1568, NCI-H1975 and NCI-H2009 were evaluated in F-azide-tagged protein pull-down, protein (re)localization analyses and for evaluation of the global effects of tipifarib. NCI-H1944 was additionally used for global effects of tipifarnib experiment. NSCLC – non-small cell lung cancer, NET – neuroendocrine tumor, CNA – copy number alteration, AUC – area under the curve, LC – lung cancer.

(B) Sequence logos for the last four amino acids ($X_0X_1X_2X_3$) at the C-terminal of all proteins with neighborhood classification (as shown in Figure 1e, *n* = 13015); proteins with a CXXX motif and either secretory (*n* = 314) or cytosolic (*n* = 414) neighborhood classification; prenylated/farnesylated proteins (PFPs identified in this study) and either secretory (*n* = 39) or cytosolic (*n* = 42) neighborhood localization. Only CXXX motifs were considered for the subsetted proteins. Enrichment *P* values were calculated using Fisher's exact test. The *P* values were then corrected for multiple hypothesis testing using false discovery rate (FDR), the resultant *q*-value < 0.05 was used as a significance cutoff. Effect size cutoff was $\log_2$ fold-enrichment > 1.

(C) Density plots of $\log_2$ fold change (FC) of protein quantity in F-azide-metabolically labeled vs control samples. FC was calculated using the DEqMS method. The number of replicates was *n* = 4 and *n* = 3 independent cell cultures for the metabolically-labeled and control samples, respectively. The background corresponds to all quantified proteins (n = 2724, 4412, 4645, and 4488 in NCI-H727, NCI-H1568, NCI-H1975, and NCI-H2009, respectively) and CXXX motif to the subset of proteins that have a CXXX motif at the C-terminal (n = 306, 446, 473 and 459, respectively).

(D) The number of overlapping enriched proteins (determined by the threshold shown in panel b) across the four cell lines. In green: candidates that have other supporting evidence (CXXX motif).

(E) Like **(D)** but the numbers are shown for the individual cell lines.

(F) Pull-down experiment results for several examples of known farnesylated proteins (CENPE, HRAS and RHEB). The data is the same as shown in panel **c** but the fold change is expressed as the percentile. Dark green: the proteins that exceeded the 95th percentile enriched protein threshold, light green: the protein did not exceed the threshold.

(G) The number of proteins with SubCellBarCode (SCBC) classification at the neighborhood level and **(H)** at the compartment level.

(I). Consistency of compartment-level classification across the cell lines.

(J) The compartment-level subcellular localization classification for proteins with a single compartment classification, *i.e.*, no conflicting classification in different cell lines.

Tipifarnib-induced protein relocalization
from the non-cytosolic to cytosolic fraction

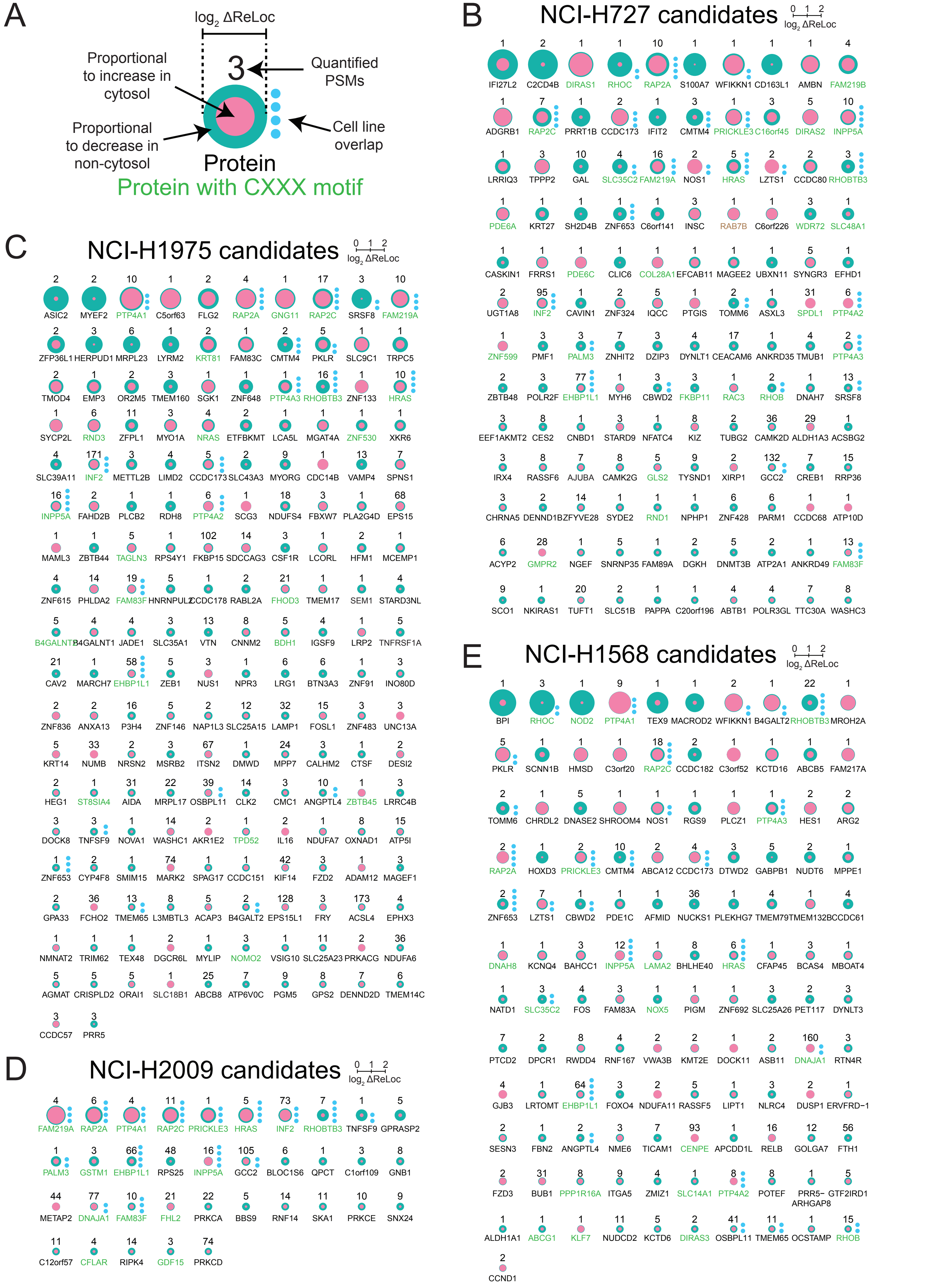

Figure S2. Tipifarnib results in widespread relocalization of proteins (related to Figure 2).

(A) Schematic illustration explaining the figure structure. ΔReLoc corresponds to the $\log_2$ fold change in the cytosolic fraction minus the $\log_2$ fold change in the non-cytosolic fraction. Criteria for relocalization: Criteria: $\log_2$ ΔReLoc > 0.4, minimum DEqMS adj. *p*-val < 0.05, opposite direction of change in cytosol and non-cytosol fractions. Proteins marked in green have a CXXX motif at the C-terminal.

(B–E) Proteins relocalizing from the non-cytosolic to the cytosolic cellular fractions after a 24-h tipifarnib treatment (1 μM) in (B) NCI-H727 cells (*n* = 130); (C) in NCI-H1975 cells (*n* = 182); (D) in NCI-H1568 cells (*n* = 121); (E) in NCI-H2009 cells (*n* = 35).

PSM – peptide-spectrum match.

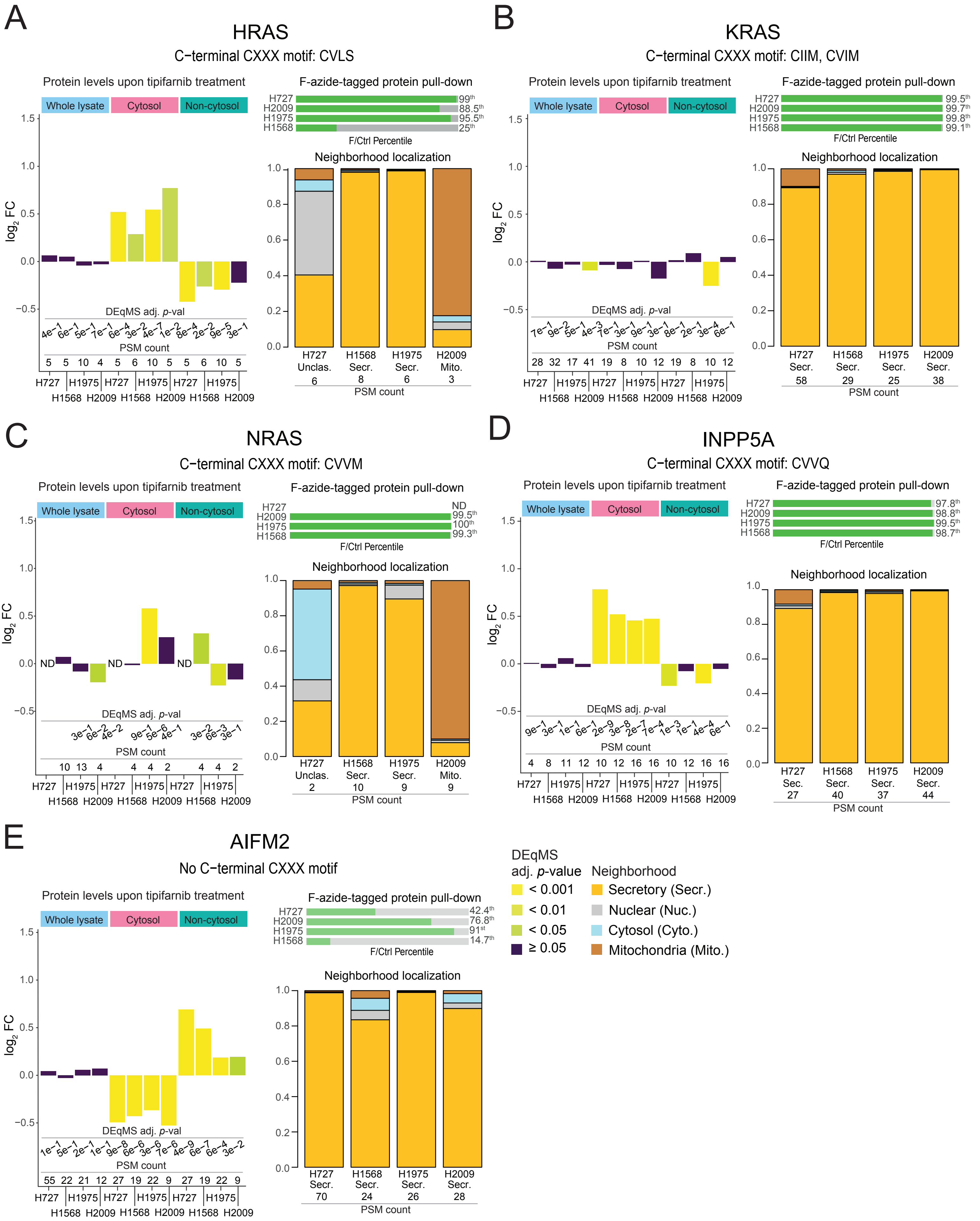

Figure S3. Examples of protein "prenylation/farnesylation passports" (related to Figures 1, 2, and 3).
(A–E) Results from the farnesylated protein pull-down experiment, protein subcellular neighborhood localization, and protein quantities after 24-h tipifarnib treatment (in the whole lysate and in cytosolic and non-cytosolic fractions). (A) HRAS. (B) KRAS. (C) NRAS. (D) INPP5A. (E) AIFM2. Also see resource R code in supplementary information.
PSM – peptide-spectrum match; ND – not detected; the NCI- prefix has been omitted in the cell line names.

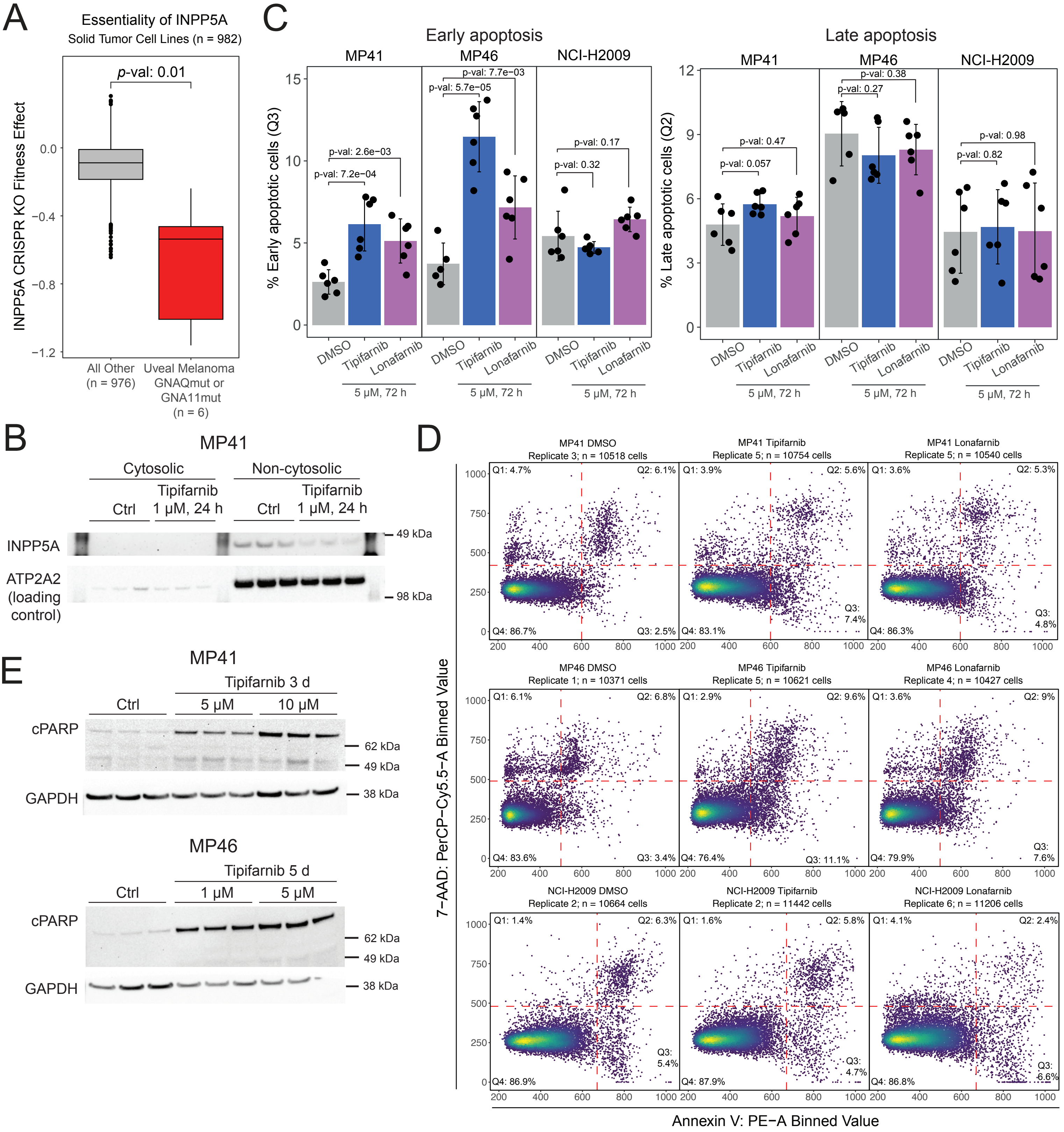
A
Essentiality of INPP5A
Solid Tumor Cell Lines (n = 982)
p-val: 0.01
INPP5A CRISPR KO Fitness Effect
0.0
−0.4
−0.8
−1.2
All Other (n = 976)
Uveal Melanoma GNAQmut or GNA11mut (n = 6)
C
Early apoptosis
MP41
MP46
NCI-H2009
% Early apoptotic cells (Q3)
p-val: 2.6e−03
p-val: 7.2e−04
p-val: 7.7e−03
p-val: 5.7e−05
p-val: 0.17
p-val: 0.32
DMSO
Tipifarnib
Lonafarnib
5 μM, 72 h
Late apoptosis
% Late apoptotic cells (Q2)
p-val: 0.47
p-val: 0.057
p-val: 0.38
p-val: 0.27
p-val: 0.98
p-val: 0.82
B
MP41
Cytosolic
Non-cytosolic
Ctrl
Tipifarnib 1 μM, 24 h
INPP5A
ATP2A2 (loading control)
49 kDa
98 kDa
D
MP41 DMSO
Replicate 3; n = 10518 cells
Q1: 4.7%
Q2: 6.1%
Q4: 86.7%
Q3: 2.5%
MP41 Tipifarnib
Replicate 5; n = 10754 cells
Q1: 3.9%
Q2: 5.6%
Q3: 7.4%
Q4: 83.1%
MP41 Lonafarnib
Replicate 5; n = 10540 cells
Q1: 3.6%
Q2: 5.3%
Q3: 4.8%
Q4: 86.3%
MP46 DMSO
Replicate 1; n = 10371 cells
Q1: 6.1%
Q2: 6.8%
Q4: 83.6%
Q3: 3.4%
MP46 Tipifarnib
Replicate 5; n = 10621 cells
Q1: 2.9%
Q2: 9.6%
Q4: 76.4%
Q3: 11.1%
MP46 Lonafarnib
Replicate 4; n = 10427 cells
Q1: 3.6%
Q2: 9%
Q3: 7.6%
Q4: 79.9%
NCI-H2009 DMSO
Replicate 2; n = 10664 cells
Q1: 1.4%
Q2: 6.3%
Q3: 5.4%
Q4: 86.9%
NCI-H2009 Tipifarnib
Replicate 2; n = 11442 cells
Q1: 1.6%
Q2: 5.8%
Q3: 4.7%
Q4: 87.9%
NCI-H2009 Lonafarnib
Replicate 6; n = 11206 cells
Q1: 4.1%
Q2: 2.4%
Q3: 6.6%
Q4: 86.8%
7−AAD: PerCP−Cy5.5−A Binned Value
Annexin V: PE−A Binned Value
E
MP41
Tipifarnib 3 d
Ctrl
5 μM
10 μM
cPARP
GAPDH
62 kDa
49 kDa
38 kDa
MP46
Tipifarnib 5 d
Ctrl
1 μM
5 μM
cPARP
GAPDH
62 kDa
49 kDa
38 kDa

Figure S4. FTIs trigger apoptosis in UVM cell lines with GNAQ/GNA11 mutations (related to Figure 2).

(A) Fitness effect of CRISPR-based INPP5A gene knockout in solid tumor cell lines. *P* value was calculated using Welch's *t*-test; the size of the groups is indicated in the figure.

(B) Western blot analysis of INPP5A in cytosolic and non-cytosolic fractions with and without tipifarnib treatment (1 μM, 24 h) in MP41 cells (*n* = 3 independent cell cultures).

(C) The number of apoptotic cells (left – early apoptosis, Q3 quadrant as shown in panel (D), right – late apoptosis, Q2 as shown in panel (D) after tipifarnib or lonafarnib treatment (5 μM, 72 h) of MP41, MP46 and NCI-H2009 cells. The values are presented as mean ± s.d. (*n* = 6 independent cell cultures, except *n* = 5 for DMSO-treated MP46 cells). *P* values (*p*-val) were calculated using two-sided two-sample *t*-test.

(D) Representative scatter plots for the apoptosis experiment presented in panel (C).

(E) Western blot analysis of cleaved PARP (cPARP) in MP41 (top) and MP46 (bottom) cells upon tipifarnib treatment for 3 and 5 days, respectively (*n* = 3 independent cell cultures). The last band in the GAPDH loading control for the MP46 experiment was accidentally cut off.

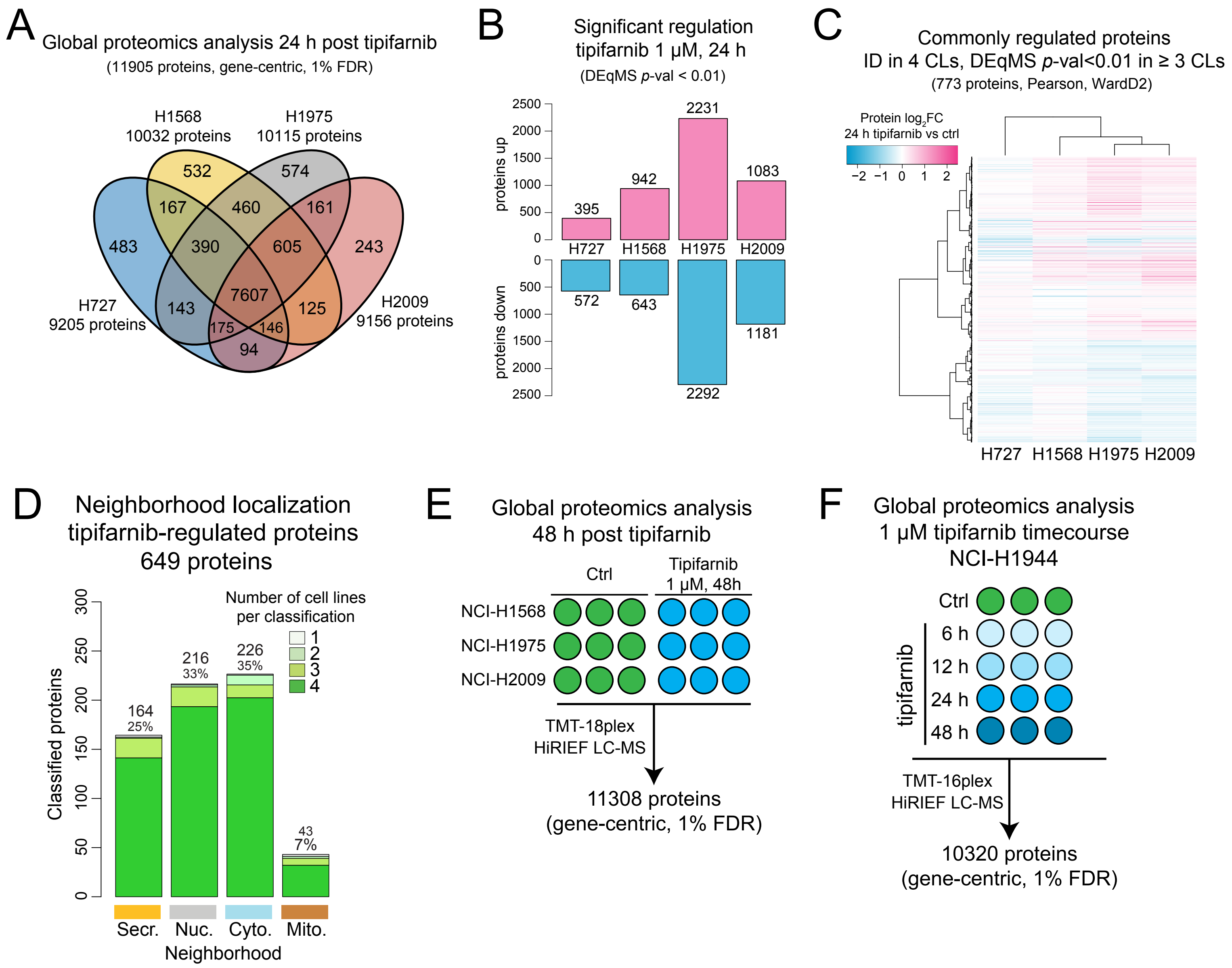

Figure S5. MS-based proteomics reveals that tipifarnib treatment leads to a complex cellular response (related to Figure 3).

(A) Venn diagram of proteins identified and quantified in NCI-H727, NCI-H1568, NCI-H1975 and NCI-H2009 cells in the 24-h 1 µM tipifarnib treatment experiment, described in Figure 1B (bottom half).

(B) Number of significantly regulated (DEqMS adj. p-value < 0.01) proteins in the 24-h 1 µM tipifarnib treatment experiment.

(C) A heatmap of the commonly regulated proteins upon the 24-h 1 µM tipifarnib treatment in the different cell lines (DEqMS adj. p-value < 0.01 in at least 3 cell lines, n = 773 proteins).

(D) Neighborhood localization of the proteins regulated by tipifarnib (1 µM) at 24 h (DEqMS adj. p-value < 0.01 in at least three cell lines).

(E-F) Experimental design of the evaluation proteome-wide effects of tipifarnib treatment at 48 h (E) and in a timecourse

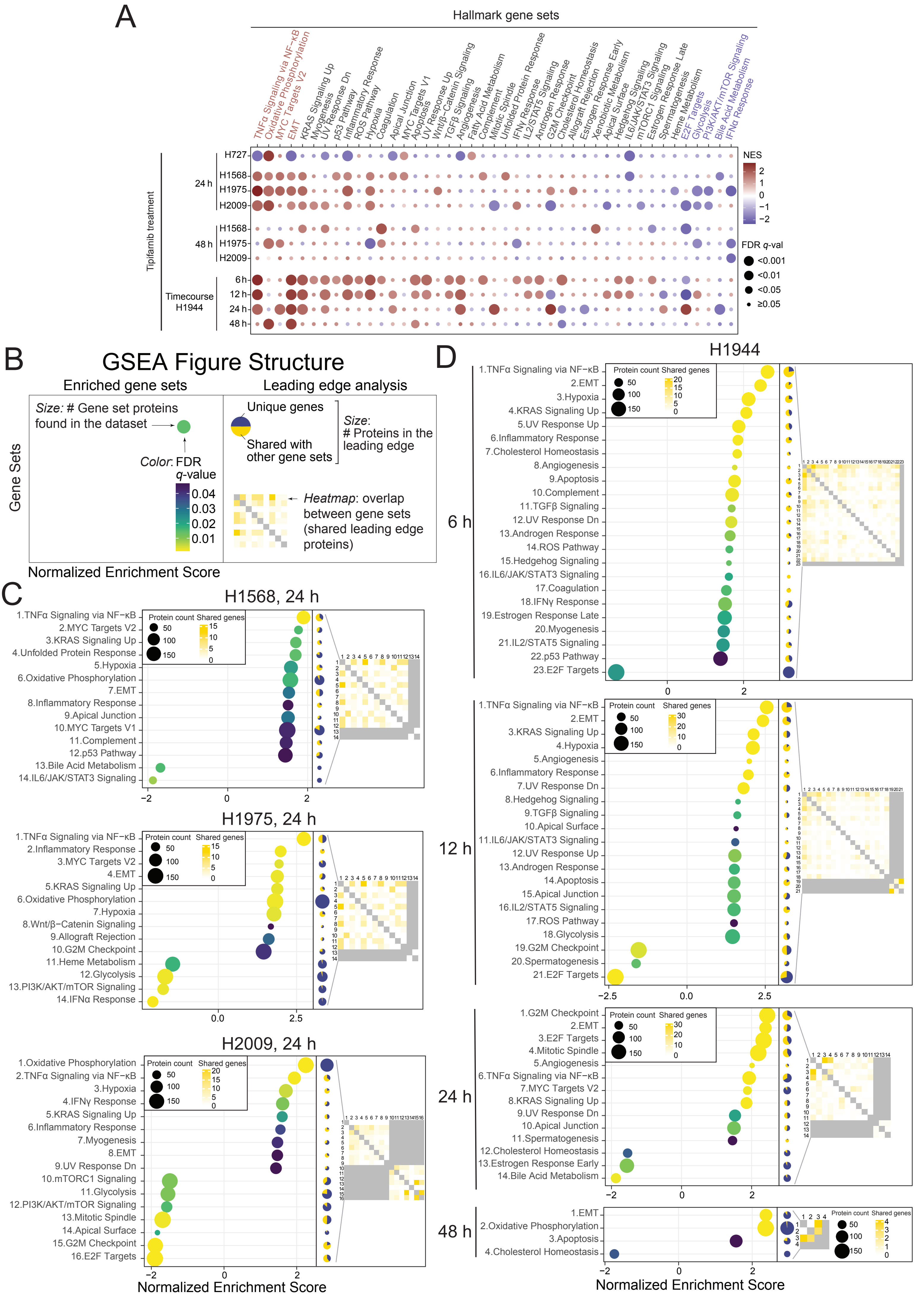
A
Hallmark gene sets
TNFα Signaling via NF−κB
Oxidative Phosphorylation
MYC Targets V2
EMT
KRAS Signaling Up
Myogenesis
UV Response Dn
p53 Pathway
Inflammatory Response
ROS Pathway
Hypoxia
Coagulation
Apical Junction
MYC Targets V1
Apoptosis
UV Response Up
Wnt/β−Catenin Signaling
TGFβ Signaling
Angiogenesis
Fatty Acid Metabolism
Complement
Mitotic Spindle
Unfolded Protein Response
IFNγ Response
IL2/STAT5 Signaling
Androgen Response
G2M Checkpoint
Cholesterol Homeostasis
Allograft Rejection
Estrogen Response Early
Xenobiotic Metabolism
Apical Surface
Hedgehog Signaling
IL6/JAK/STAT3 Signaling
mTORC1 Signaling
Estrogen Response Late
Spermatogenesis
Heme Metabolism
E2F Targets
Glycolysis
PI3K/AKT/mTOR Signaling
Bile Acid Metabolism
IFNα Response
Tipifarnib treatment
24 h
H727
H1568
H1975
H2009
48 h
H1568
H1975
H2009
Timecourse H1944
6 h
12 h
24 h
48 h
NES
2
1
0
−1
−2
FDR q-val
<0.001
<0.01
<0.05
≥0.05
B
GSEA Figure Structure
Enriched gene sets
Leading edge analysis
Gene Sets
Size: # Gene set proteins found in the dataset
Color: FDR q-value
0.04
0.03
0.02
0.01
Unique genes
Shared with other gene sets
Size: # Proteins in the leading edge
Heatmap: overlap between gene sets (shared leading edge proteins)
Normalized Enrichment Score
C
H1568, 24 h
1.TNFα Signaling via NF−κB
2.MYC Targets V2
3.KRAS Signaling Up
4.Unfolded Protein Response
5.Hypoxia
6.Oxidative Phosphorylation
7.EMT
8.Inflammatory Response
9.Apical Junction
10.MYC Targets V1
11.Complement
12.p53 Pathway
13.Bile Acid Metabolism
14.IL6/JAK/STAT3 Signaling
Protein count Shared genes
50
100
150
15
10
5
0
−2
0
2
H1975, 24 h
1.TNFα Signaling via NF−κB
2.Inflammatory Response
3.MYC Targets V2
4.EMT
5.KRAS Signaling Up
6.Oxidative Phosphorylation
7.Hypoxia
8.Wnt/β−Catenin Signaling
9.Allograft Rejection
10.G2M Checkpoint
11.Heme Metabolism
12.Glycolysis
13.PI3K/AKT/mTOR Signaling
14.IFNα Response
0.0
2.5
H2009, 24 h
1.Oxidative Phosphorylation
2.TNFα Signaling via NF−κB
3.Hypoxia
4.IFNγ Response
5.KRAS Signaling Up
6.Inflammatory Response
7.Myogenesis
8.EMT
9.UV Response Dn
10.mTORC1 Signaling
11.Glycolysis
12.PI3K/AKT/mTOR Signaling
13.Mitotic Spindle
14.Apical Surface
15.G2M Checkpoint
16.E2F Targets
Protein count Shared genes
20
15
10
5
0
−2
0
2
Normalized Enrichment Score
D
H1944
6 h
1.TNFα Signaling via NF−κB
2.EMT
3.Hypoxia
4.KRAS Signaling Up
5.UV Response Up
6.Inflammatory Response
7.Cholesterol Homeostasis
8.Angiogenesis
9.Apoptosis
10.Complement
11.TGFβ Signaling
12.UV Response Dn
13.Androgen Response
14.ROS Pathway
15.Hedgehog Signaling
16.IL6/JAK/STAT3 Signaling
17.Coagulation
18.IFNγ Response
19.Estrogen Response Late
20.Myogenesis
21.IL2/STAT5 Signaling
22.p53 Pathway
23.E2F Targets
0
2
12 h
1.TNFα Signaling via NF−κB
2.EMT
3.KRAS Signaling Up
4.Hypoxia
5.Angiogenesis
6.Inflammatory Response
7.UV Response Dn
8.Hedgehog Signaling
9.TGFβ Signaling
10.Apical Surface
11.IL6/JAK/STAT3 Signaling
12.UV Response Up
13.Androgen Response
14.Apoptosis
15.Apical Junction
16.IL2/STAT5 Signaling
17.ROS Pathway
18.Glycolysis
19.G2M Checkpoint
20.Spermatogenesis
21.E2F Targets
30
20
10
0
−2.5
0.0
2.5
24 h
1.G2M Checkpoint
2.EMT
3.E2F Targets
4.Mitotic Spindle
5.Angiogenesis
6.TNFα Signaling via NF−κB
7.MYC Targets V2
8.KRAS Signaling Up
9.UV Response Dn
10.Apical Junction
11.Spermatogenesis
12.Cholesterol Homeostasis
13.Estrogen Response Early
14.Bile Acid Metabolism
−2
0
2
48 h
1.EMT
2.Oxidative Phosphorylation
3.Apoptosis
4.Cholesterol Homeostasis
4
3
2
1
0
−2
0
2
Normalized Enrichment Score

Figure S6. MS-based proteomics reveals that tipifarnib treatment leads to a complex cellular response (related to Figure 3).

(A) Overview of all GSEA enrichment normalized enrichment scores (NES).

(B) Schematic illustration of the figure structure for detailed GSEA plots, including leading edge analysis.

(C) Enriched gene sets (*q*-value < 0.05) in datasets for 24-h tipifarnib treatment in NCI-H1568, NCI-H1975, and NCI-H2009 cells.

(D) Enriched gene sets (*q*-value < 0.05) in datasets for a timecourse tipifarnib treatment in NCI-H1944 cells. The

NCI- prefix has been omitted in the cell line names

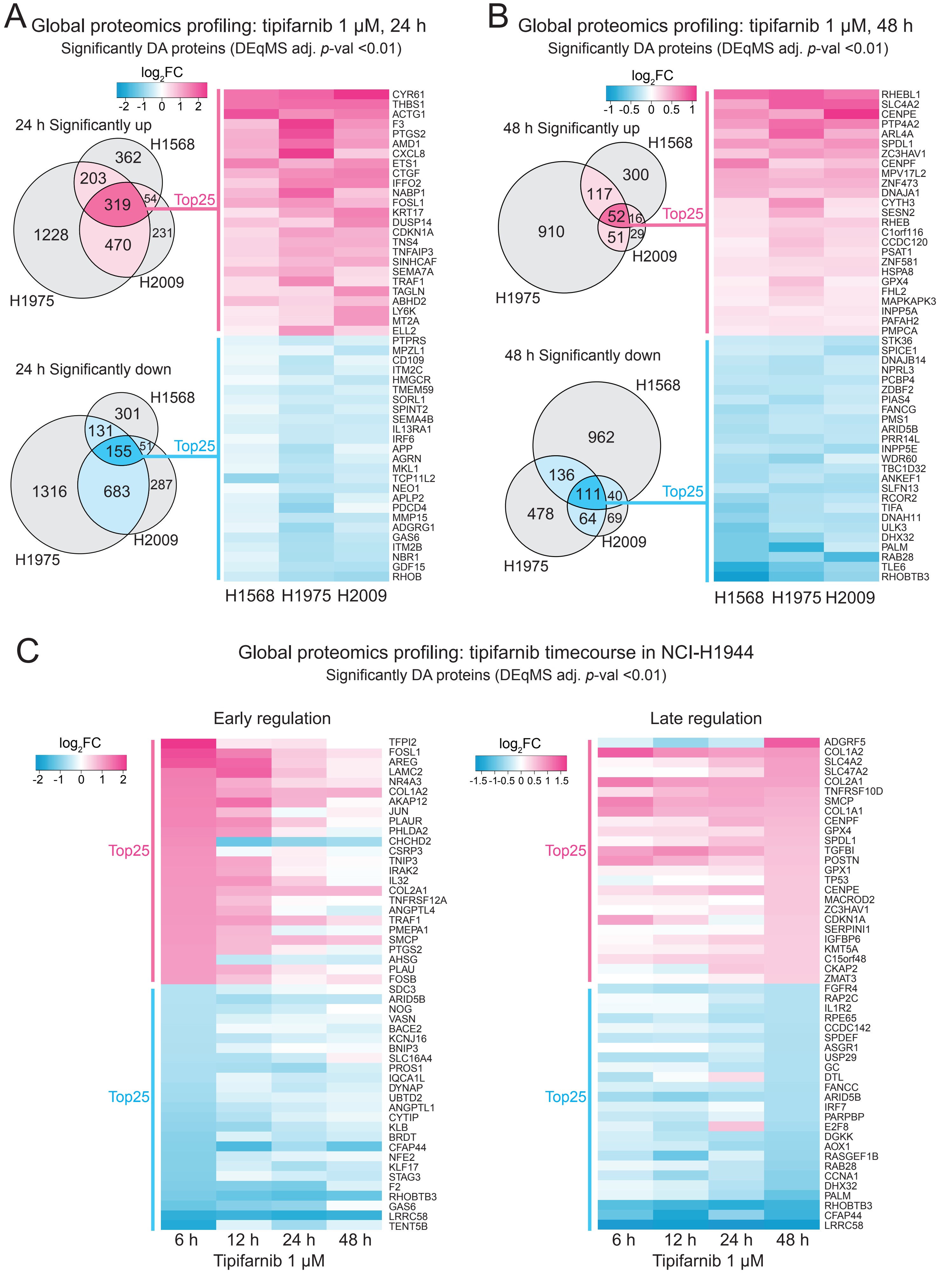
A Global proteomics profiling: tipifarnib 1 µM, 24 h
Significantly DA proteins (DEqMS adj. p-val <0.01)
log2FC
-2 -1 0 1 2
24 h Significantly up
H1568
362
203
319
54
Top25
1228
470
231
H2009
H1975
24 h Significantly down
H1568
301
131
155
51
Top25
1316
683
287
H2009
H1975
CYR61
THBS1
ACTG1
F3
PTGS2
AMD1
CXCL8
ETS1
CTGF
IFFO2
NABP1
FOSL1
KRT17
DUSP14
CDKN1A
TNS4
TNFAIP3
SINHCAF
SEMA7A
TRAF1
TAGLN
ABHD2
LY6K
MT2A
ELL2
PTPRS
MPZL1
CD109
ITM2C
HMGCR
TMEM59
SORL1
SPINT2
SEMA4B
IL13RA1
IRF6
APP
AGRN
MKL1
TCP11L2
NEO1
APLP2
PDCD4
MMP15
ADGRG1
GAS6
ITM2B
NBR1
GDF15
RHOB
H1568 H1975 H2009
B Global proteomics profiling: tipifarnib 1 µM, 48 h
Significantly DA proteins (DEqMS adj. p-val <0.01)
log2FC
-1 -0.5 0 0.5 1
48 h Significantly up
H1568
300
117
52
16
Top25
910
51
29
H2009
H1975
48 h Significantly down
H1568
962
136
111
40
Top25
478
64
69
H2009
H1975
RHEBL1
SLC4A2
CENPE
PTP4A2
ARL4A
SPDL1
ZC3HAV1
CENPF
MPV17L2
ZNF473
DNAJA1
CYTH3
SESN2
RHEB
C1orf116
CCDC120
PSAT1
ZNF581
HSPA8
GPX4
FHL2
MAPKAPK3
INPP5A
PAFAH2
PMPCA
STK36
SPICE1
DNAJB14
NPRL3
PCBP4
ZDBF2
PIAS4
FANCG
PMS1
ARID5B
PRR14L
INPP5E
WDR60
TBC1D32
ANKEF1
SLFN13
RCOR2
TIFA
DNAH11
ULK3
DHX32
PALM
RAB28
TLE6
RHOBTB3
H1568 H1975 H2009
C Global proteomics profiling: tipifarnib timecourse in NCI-H1944
Significantly DA proteins (DEqMS adj. p-val <0.01)
Early regulation
log2FC
-2 -1 0 1 2
Top25
Top25
TFPI2
FOSL1
AREG
LAMC2
NR4A3
COL1A2
AKAP12
JUN
PLAUR
PHLDA2
CHCHD2
CSRP3
TNIP3
IRAK2
IL32
COL2A1
TNFRSF12A
ANGPTL4
TRAF1
PMEPA1
SMCP
PTGS2
AHSG
PLAU
FOSB
SDC3
ARID5B
NOG
VASN
BACE2
KCNJ16
BNIP3
SLC16A4
PROS1
IQCA1L
DYNAP
UBTD2
ANGPTL1
CYTIP
KLB
BRDT
CFAP44
NFE2
KLF17
STAG3
F2
RHOBTB3
GAS6
LRRC58
TENT5B
6 h 12 h 24 h 48 h
Tipifarnib 1 µM
Late regulation
log2FC
-1.5 -1 -0.5 0 0.5 1 1.5
Top25
Top25
ADGRF5
COL1A2
SLC4A2
SLC47A2
COL2A1
TNFRSF10D
SMCP
COL1A1
CENPF
GPX4
SPDL1
TGFBI
POSTN
GPX1
TP53
CENPE
MACROD2
ZC3HAV1
CDKN1A
SERPINI1
IGFBP6
KMT5A
C15orf48
CKAP2
ZMAT3
FGFR4
RAP2C
IL1R2
RPE65
CCDC142
SPDEF
ASGR1
USP29
GC
DTL
FANCC
ARID5B
IRF7
PARPBP
E2F8
DGKK
AOX1
RASGEF1B
RAB28
CCNA1
DHX32
PALM
RHOBTB3
CFAP44
LRRC58
6 h 12 h 24 h 48 h
Tipifarnib 1 µM

Figure S7. Top up and down regulated proteins in response to tipifarnib treatment (related to Figure 3).

(A–C) Heatmaps showing the protein quantities of the most regulated (significantly differentially abundant, DA) proteins upon tipifarnib treatment (25 highest and 25 lowest log_2 fold change (FC) values vs ctrl, DEqMS adj. *p*-val < 0.01) and Venn diagrams showing their overlap in the different cell lines upon (A) 24-h tipifarnib treatment; (B) 48-h treatment; (C) timecourse experiment in NCI-H1944 cells. Early regulation is defined as the top differentially abundant (DA) proteins at 6 h and later regulation at 48 h.

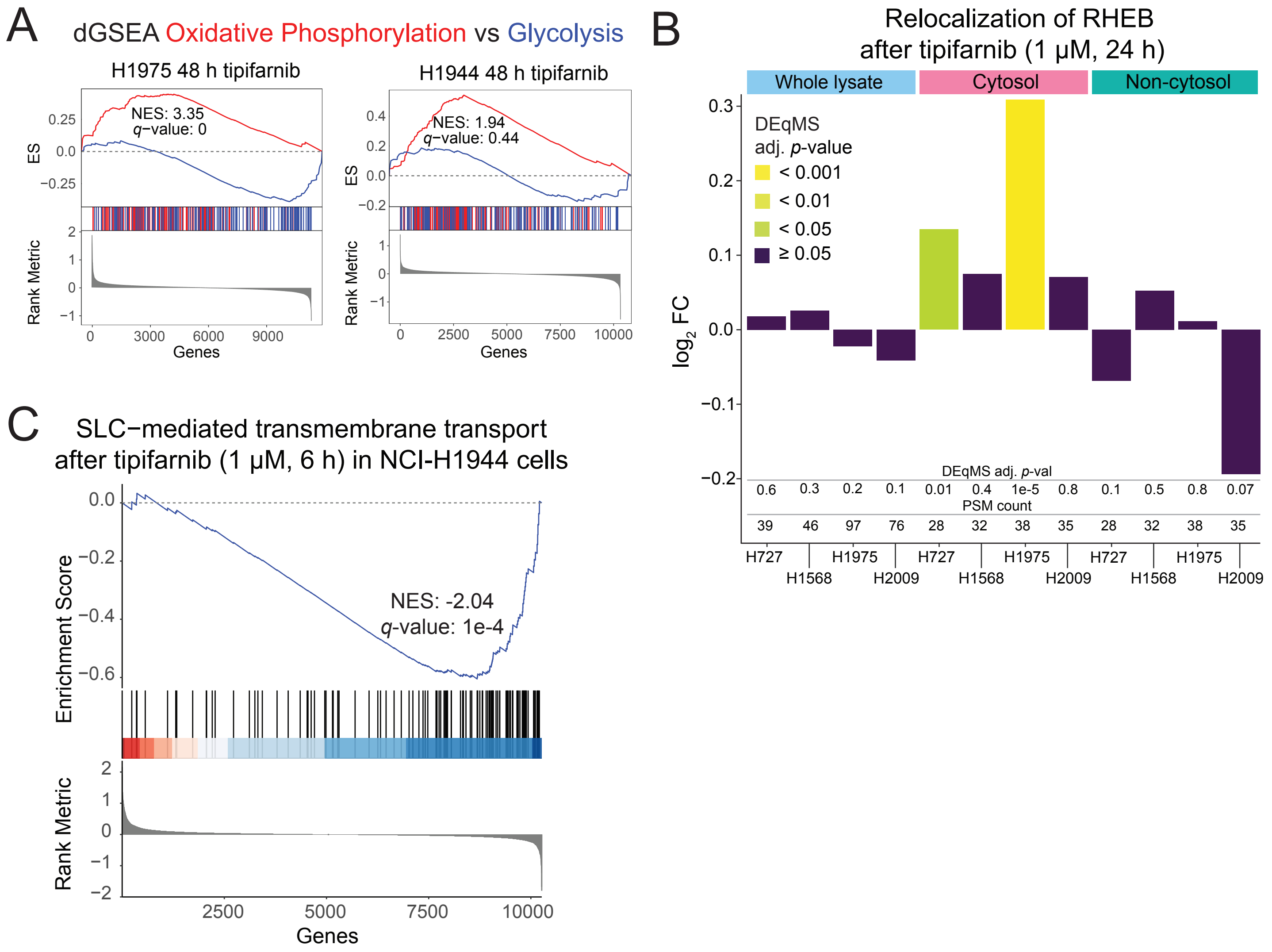

Figure S8. Global effects of tipifarnib treatment (related to Figure 3).

(A) Differential gene set enrichment analysis (dGSEA) comparing "hallmark" gene sets oxidative phosphorylation and glycolysis upon 48-h tipifarnib treatment in NCI-H1944 cells. (N)ES – (normalized) enrichment score.

(B) Quantities of RHEB in the whole cell lysate, and in cytosolic and non-cytosolic fractions, expressed as $\log_2$ fold change (FC) in tipifarnib-treated (24 h) vs ctrl cells. PSM – peptide-spectrum match.

(C) Gene set enrichment plot of SLC-mediated transmembrane transport in NCI-H1944 cells after a 6-h tipifarnib treatment. The NCI- prefix has been omitted in the cell line names.

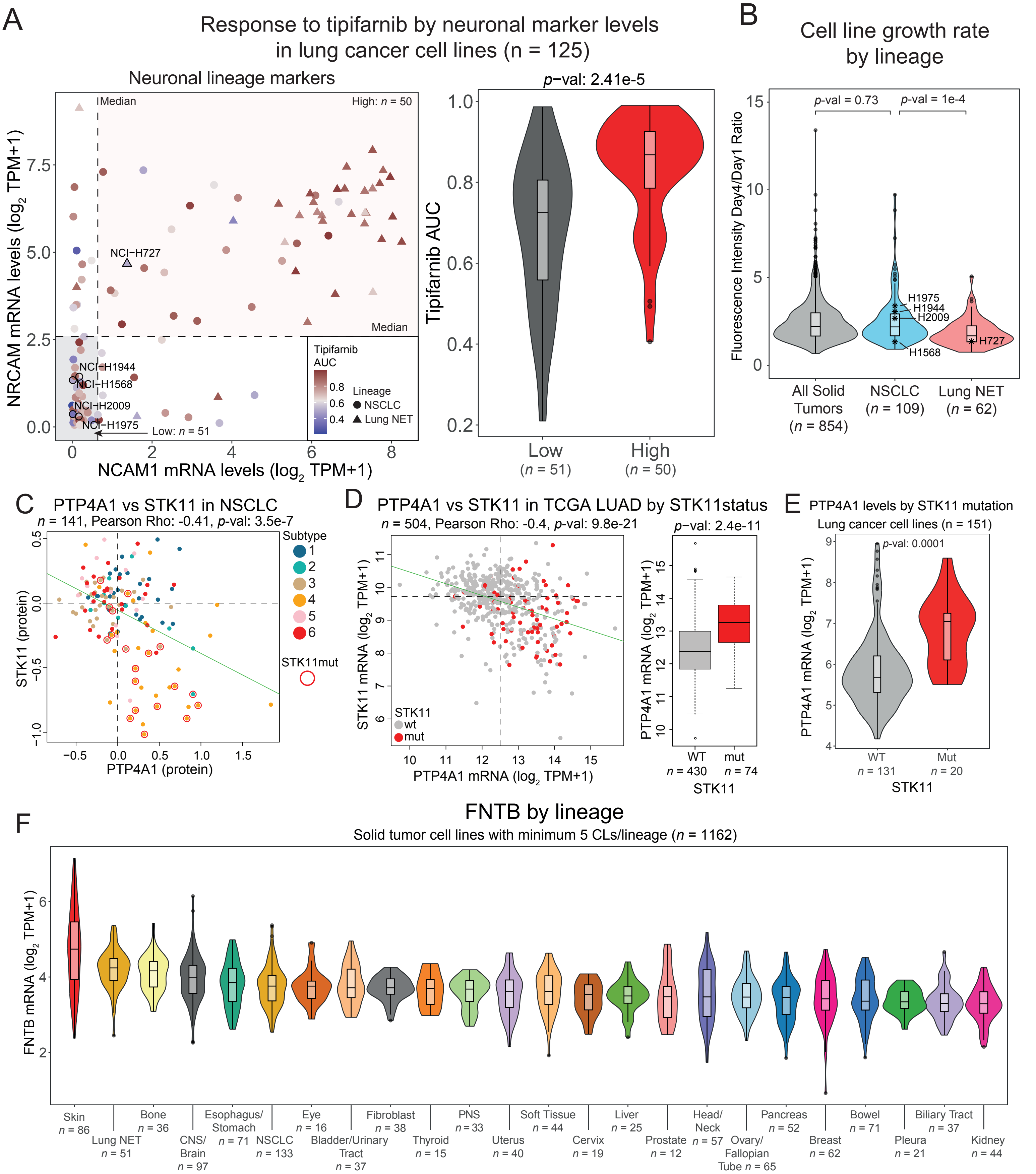

Figure S9. Publicly available data analysis (related to Figure 4).

(A) NCAM1 and NRCAM mRNA levels (from DepMap portal) in lung cancer cell lines (n = 125), colored by response to tipifarnib (AUC – area under the curve). And a comparison of response to tipifarnib in cell lines with high NCAM1 and NRCAM levels (both above median, n = 50) vs low levels (both below median, n = 51). The *P* value was calculated using Welch t-test. NSCLC – non-small cell lung cancer, NET – neuroendocrine tumor.

(B) Cell line growth rate in solid tumor cell lines, NSCLC cell lines and lung NET cell lines (from Cell Model Passport database). *P* values were calculated using Welch t-test, the number of cell lines per group are indicated in the figure.

(C) PTP4A1 and STK11 proteins levels in NSCLC cohort published in Lehtiö *et al*. (2021).

(D) PTP4A1 and STK11 mRNA levels in lung adenocarcinoma (LUAD) published in TCGA and PTP4A1 mRNA levels by STK11 mutation status.

(E). PTP4A1 mRNA levels (from DepMap portal) in lung cancer cell lines by STK11 mutation status. The *P* value was calculated using Welch t-test.

(F) FNTB mRNA levels (from DepMap portal) in solid tumor cell lines by lineage.

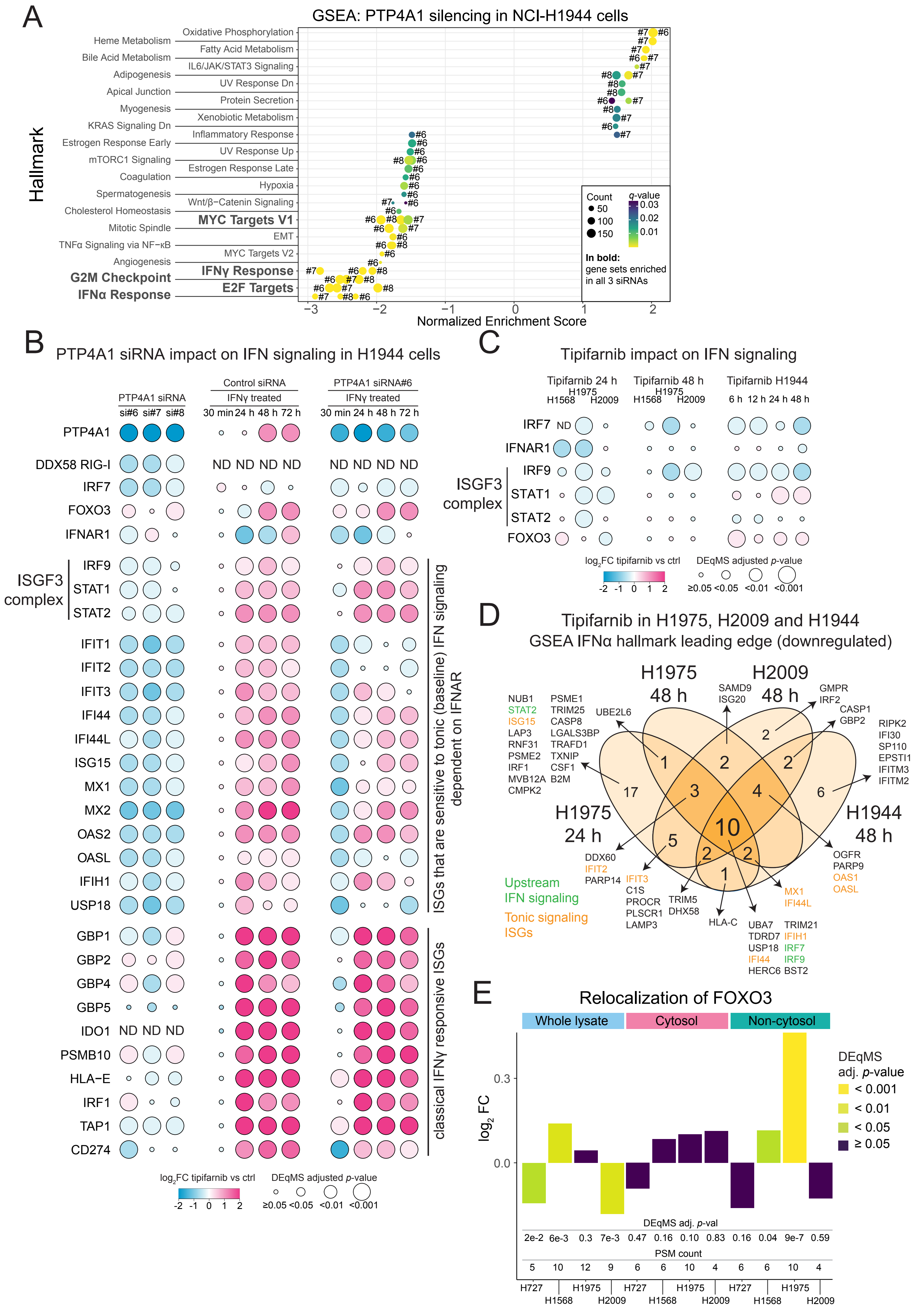
A
GSEA: PTP4A1 silencing in NCI-H1944 cells
Hallmark
Oxidative Phosphorylation
Heme Metabolism
Fatty Acid Metabolism
Bile Acid Metabolism
IL6/JAK/STAT3 Signaling
Adipogenesis
UV Response Dn
Apical Junction
Protein Secretion
Myogenesis
Xenobiotic Metabolism
KRAS Signaling Dn
Inflammatory Response
Estrogen Response Early
UV Response Up
mTORC1 Signaling
Estrogen Response Late
Coagulation
Hypoxia
Spermatogenesis
Wnt/β−Catenin Signaling
Cholesterol Homeostasis
MYC Targets V1
Mitotic Spindle
EMT
TNFα Signaling via NF−κB
MYC Targets V2
Angiogenesis
IFNγ Response
G2M Checkpoint
E2F Targets
IFNα Response
Count
50
100
150
q-value
0.03
0.02
0.01
In bold:
gene sets enriched
in all 3 siRNAs
−3
−2
−1
0
1
2
Normalized Enrichment Score
B
PTP4A1 siRNA impact on IFN signaling in H1944 cells
PTP4A1 siRNA
si#6 si#7 si#8
Control siRNA
IFNγ treated
30 min 24 h 48 h 72 h
PTP4A1 siRNA#6
IFNγ treated
30 min 24 h 48 h 72 h
PTP4A1
DDX58 RIG-I
IRF7
FOXO3
IFNAR1
ISGF3 complex
IRF9
STAT1
STAT2
IFIT1
IFIT2
IFIT3
IFI44
IFI44L
ISG15
MX1
MX2
OAS2
OASL
IFIH1
USP18
GBP1
GBP2
GBP4
GBP5
IDO1
PSMB10
HLA−E
IRF1
TAP1
CD274
ND
ISGs that are sensitive to tonic (baseline) IFN signaling dependent on IFNAR
classical IFNγ responsive ISGs
log2FC tipifarnib vs ctrl
-2 -1 0 1 2
DEqMS adjusted p-value
≥0.05 <0.05 <0.01 <0.001
C
Tipifarnib impact on IFN signaling
Tipifarnib 24 h
H1568 H1975 H2009
Tipifarnib 48 h
H1568 H1975 H2009
Tipifarnib H1944
6 h 12 h 24 h 48 h
IRF7
IFNAR1
ISGF3 complex
IRF9
STAT1
STAT2
FOXO3
ND
log2FC tipifarnib vs ctrl
-2 -1 0 1 2
DEqMS adjusted p-value
≥0.05 <0.05 <0.01 <0.001
D
Tipifarnib in H1975, H2009 and H1944
GSEA IFNα hallmark leading edge (downregulated)
H1975 48 h
H2009 48 h
H1975 24 h
H1944 48 h
17
1
2
2
6
3
4
10
5
2
2
1
NUB1
STAT2
ISG15
LAP3
RNF31
PSME2
IRF1
MVB12A
CMPK2
PSME1
TRIM25
CASP8
LGALS3BP
TRAFD1
TXNIP
CSF1
B2M
UBE2L6
SAMD9
ISG20
GMPR
IRF2
CASP1
GBP2
RIPK2
IFI30
SP110
EPSTI1
IFITM3
IFITM2
DDX60
IFIT2
PARP14
IFIT3
C1S
PROCR
PLSCR1
LAMP3
TRIM5
DHX58
HLA-C
UBA7
TDRD7
USP18
IFI44
HERC6
TRIM21
IFIH1
IRF7
IRF9
BST2
MX1
IFI44L
OGFR
PARP9
OAS1
OASL
Upstream
IFN signaling
Tonic signaling
ISGs
E
Relocalization of FOXO3
Whole lysate
Cytosol
Non-cytosol
log2 FC
0.3
0.0
DEqMS
adj. p-value
< 0.001
< 0.01
< 0.05
≥ 0.05
DEqMS adj. p-val
2e-2 6e-3 0.3 7e-3 0.47 0.16 0.10 0.83 0.16 0.04 9e-7 0.59
PSM count
5 10 12 9 6 6 10 4 6 6 10 4
H727 H1568 H1975 H2009 H727 H1568 H1975 H2009 H727 H1568 H1975 H2009

Figure S10. Proteomics analysis implicates PTP4A1 in regulation of tonic IFN signaling.

(A) Gene set enrichment analysis (GSEA) of PTP4A1 silencing using three different siRNAs. Only significantly enriched "hallmark" gene sets are presented (*q*-value < 0.05). Count refers to the number of gene set proteins identified in the dataset. EMT – epithelial mesenchymal transition

(B). IFN signaling proteins upon PTP4A1 silencing and/or IFNγ treatment (10 ng/mL), *n* = 3 independent cell cultures. The experimental design is displayed in Figures 5D and 5H. Fold change (FC) and adjusted *P* values (adj. *p*-val) were calculated using the DEqMS method. ISGs – interferon-stimulated genes.

(C) IFN signaling proteins upon tipifarnib treatment (1 µM), the experimental designs are displayed in Figure 1B and Figures S5E and S5F. Fold change (FC) and adjusted *P* values (adj. *p*-val) were calculated using the DEqMS method.

(D) Proteins part of the gene set enrichment analysis (GSEA) leading edge of IFN hallmark gene set.

(E) FOXO3 protein levels in the whole cell lysate, and in cytosolic and non-cytosolic fractions after a 24-h tipifarnib treatment (1 µM). Fold change (FC) and adjusted *P* values (adj. *p*-val) were calculated using the DEqMS method. The experimental design is displayed in Figure 2A. PSM – peptide-spectrum match.

In panels **(B)** and **(C)**: ND – not detected.

Viability of Cells (%) upon Treatment with Tipifarnib combined with RSL3

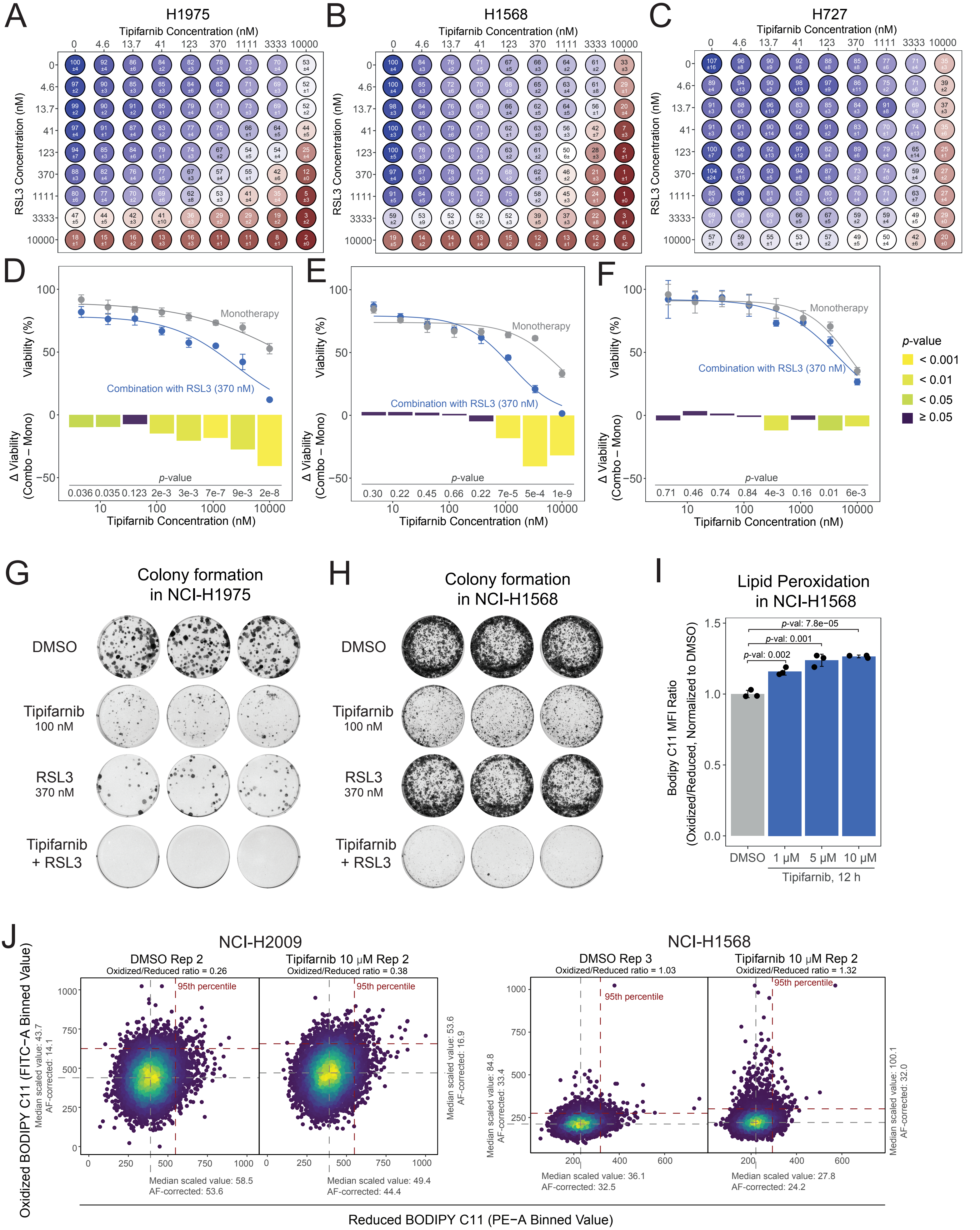

Figure S11. Tipifarnib triggers ferroptosis and is synergistic with a ferroptosis inducer RSL3 in a subset of cell lines (related to Figure 6)

(A–C) Viability of cells upon tipifarnib and/or ferroptosis inducer RSL3 treatment evaluated in a 72-h CellTiter-Glo-based assay in a 384-well plate format in (A) NCI-H1975, (B) NCI-H1568, and (C) NCI-H717 cells. The values presented are mean ± s.d (*n* = 8 wells for drug monotherapies and *n* = 3 wells for drug combinations).

(D–F) Drug response curves derived from the data presented in panels (A–C) with regression lines derived using nls() function in R, displayed for (D) NCI-H2009, (E) NCI-H1568 and (F) NCI-H727 cells. The differences between the viability upon monotherapy (Mono) and drug combination (Combo) treatment were calculated and the two-sided *t*-test *P* values (*p*-val) are presented.

(G–H). Colony formation in (G) NCI-H1975 and (H) NCI-H1568 cells treated with tipifarnib and RSL3 alone or in combination for 11 days. *n* = 3 wells were performed, all are shown.

(I). Lipid peroxidation in NCI-H1568 cells measured as the ratio of oxidized to reduced BODIPY C11 autofluorescence-corrected median fluorescence intensity (MFI). The values were normalized to the DMSO control and presented as mean ± s.d. (*n* = 3 independent cell cultures).

(J). Representative scatter plots from the lipid peroxidation experiment presented in panel (I) and Figure 6F. Flowjo binned values were used for plotting, the statistics presented in panel (I) and Figure 6F were performed on the scaled values (the corresponding median values are indicated in the scatter plots).

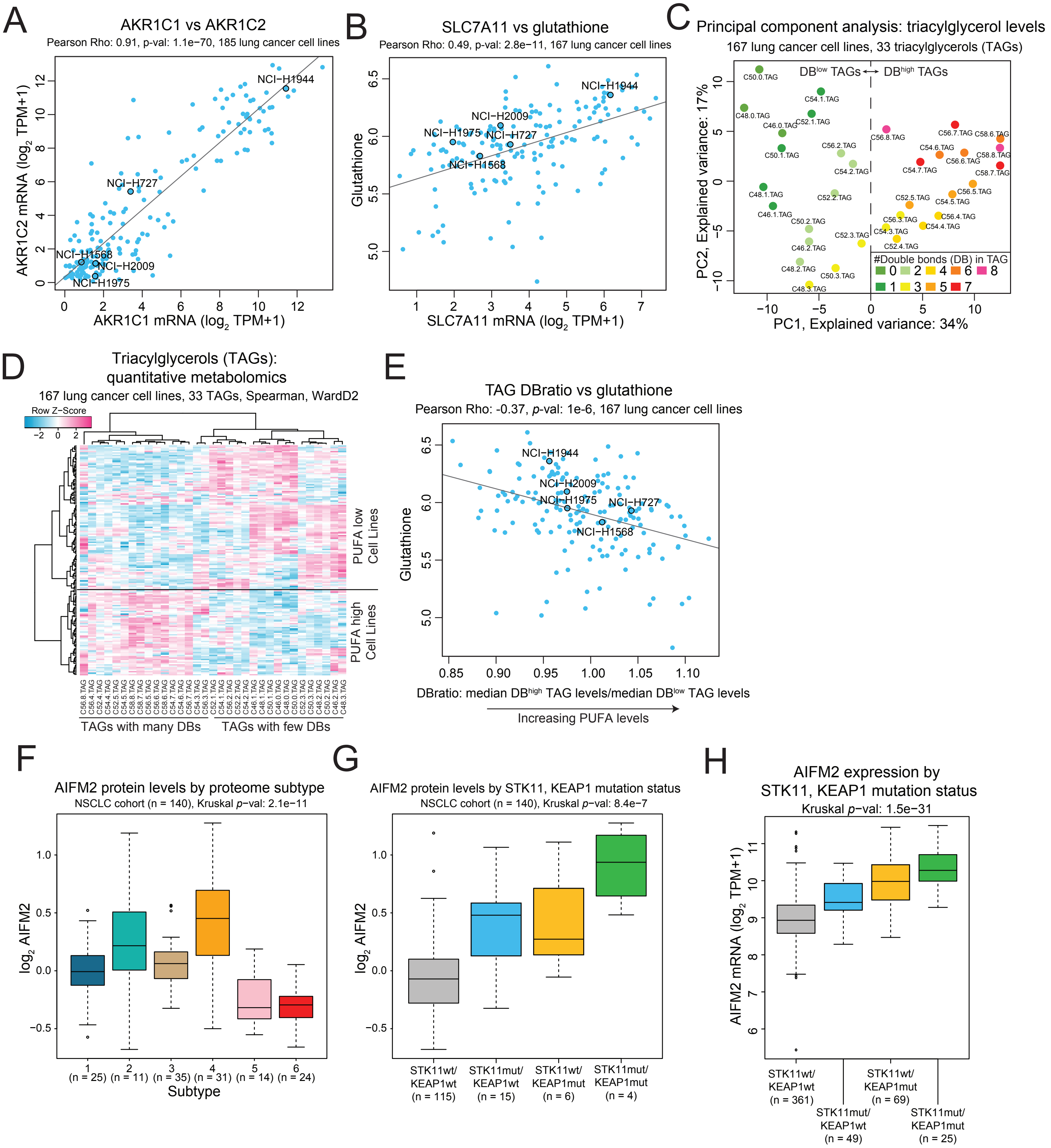

Figure S12. STK11/KEAP1 mutations modify response to ferroptosis inducers.

(A) AKR1C1 and AKR1C2 mRNA levels (from DepMap portal) in lung cancer cell lines.

(B) SLC7A11 mRNA levels (from DepMap portal) and glutathione levels (from Li *et al.*, 2019) in lung cancer cell lines.

(C) Principal component analysis based on quantitative metabolomics data (from Li *et al.*, 2019) for triacylglycerols (TAGs), colored by the number of double bonds (DBs) in the TAG.

(D) Quantitative metabolomics data for triacylglycerols from Li *et al.*, 2019.

(E) TAG DB ratio was calculated for each cell line by dividing the median levels of TAGs with a high number of DBs (DBhigh TAGs as indicated in panel **(C)** by the median levels of TAGs with a low number of DBs (DBlow TAGs as indicated in panel **(C)** TAG DBratio was then plotted again glutathione levels (from Li *et al.*, 2019).

(F) AIFM2 proteins levels in the Lehtiö *et al*. (2021) NSCLC cohort. *P* value was calculated using Kruskal-Wallis test and the number of samples per subtype is indicated.

(G) AIFM2 proteins levels by STK11 and KEAP1 mutation status in the Lehtiö *et al*. (2021) NSCLC cohort. *P* value was calculated using Kruskal-Wallis test and the number of samples per subtype is indicated.

(H) AIFM2 mRNA levels (from TCGA) in lung adenocarcinoma (LUAD) by STK11 and KEAP1 mutation status.
